# Factors affecting communication during telephone triage in medical call centres: a mixed methods systematic review

**DOI:** 10.1186/s13643-024-02580-7

**Published:** 2024-06-22

**Authors:** Siri-Linn Schmidt Fotland, Vivian Midtbø, Jorunn Vik, Erik Zakariassen, Ingrid Hjulstad Johansen

**Affiliations:** 1 National Centre for Emergency Primary Health Care, NORCE Norwegian Research Centre AS, Box 22, Bergen, NO-5838 Norway; 2 Department of Global Public Health and Primary Care, Faculty of Medicine, University of Bergen, Box 7804, Bergen, NO-5020 Norway; 3 The Regional Centre for Emergency Medical Research and Development in Western Norway (RAKOS), Stavanger University Hospital, Box 8100, Stavanger, NO-4068 Norway

**Keywords:** Telephone triage, Telenursing, Emergency Medical Services, Out-of-hours medical care, Communication

## Abstract

**Background:**

Telephone triage is used to optimise patient flow in emergency primary healthcare. Poor communication can lead to misunderstandings and compromise patient safety. To improve quality, a comprehensive understanding of factors affecting communication in medical call centres in primary care is needed. The aim of this review was to identify such factors and to describe how they affect communication during telephone triage.

**Method:**

A mixed-method systematic review was performed. In April 2021 and June 2023, MEDLINE, Embase, CINAHL, and Web of Science were searched for original studies describing communication during telephone triage in primary care medical call centres handling all types of medical problems from an unselected population. All studies were screened by two authors, blinded to each other’s decisions. Disagreements were resolved by a third author. A framework was created by the thematic synthesis of the qualitative data and later used to synthesise the quantitative data. By using convergent integrated synthesis, the qualitative and quantitative findings were integrated. The Mixed Methods Appraisal Tool was used to assess methodological limitations.

**Results:**

Out of 5087 studies identified in the search, 62 studies were included, comprising 40 qualitative, 16 quantitative and six mixed-method studies. Thirteen factors were identified and organised into four main themes: organisational factors, factors related to the operator, factors related to the caller and factors in the interaction. Organisational factors included availability, working conditions and decision support systems. Factors related to the operator were knowledge and experience, personal qualities and communication strategies. Factors related to the caller were individual differences and the presented medical problem. Factors in the interaction were faceless communication, connection between operator and caller, third-person caller and communication barriers. The factors seem interrelated, with organisational factors affecting all parts of the conversation, and the operator’s communication in particular.

**Conclusion:**

Many factors affect the structure, content, and flow of the conversation. The operators influence the communication directly but rely on the organisation to create a working environment that facilitates good communication. The results are mainly supported by qualitative studies and further studies are needed to explore and substantiate the relevance and effect of individual factors.

**Systematic review registration:**

PROSPERO CRD42022298022.

**Supplementary Information:**

The online version contains supplementary material available at 10.1186/s13643-024-02580-7.

## Background

Telephone triage is increasingly used to navigate patients to the appropriate level of care and manage the patient flow [1, 2]. Telephone triage involves assessing the patient’s symptoms, determining the level of urgency and type of healthcare needed and providing self-care advice, if appropriate. A review shows that approximately 50% of the calls handled by nurses or doctors in medical call centres in primary care can be handled with self-care advice alone [3], consequently reducing the pressure on the services [4].

The same review also concluded that there is no greater safety concern with telephone triage than with traditional face-to-face care [3]. However, studies show that the accuracy of decisions is positively associated with high-quality communication [5, 6], and conversely, inadequate communication can reduce patient safety [7–10]. In addition, the caller’s satisfaction with the conversation is associated with an increased likelihood of following the medical advice given [11]. Thus, ensuring good communication is a way of ensuring patient safety and a good quality of the healthcare delivered.

Good communication in telephone triage has been described in various communication assessment tools developed via Delphi processes and with professional grounding [12–14]. While studies that have evaluated actual calls show noticeable variations in quality [5, 6, 15], limited attention has been directed to exploring the factors that contribute to the observed variation. Therefore, this review aims to identify factors affecting communication during telephone triage in emergency primary healthcare and to describe how these factors affect communication.

## Methods

### Design

A mixed-method systematic review was chosen to encompass findings from all types of primary studies. The review was performed using the 24-step guide for systematic review published by Muka et al. [16] as a guideline and reported according to the Preferred Reporting Items for Systematic Review and Meta-Analyses (PRISMA) checklist [17]. The checklist is available in Additional file 1. The study protocol was registered in PROSPERO January 2021 (CRD42022298022).

### Eligibility criteria

To identify and describe the research question, we used the SPIDER methodology (Sample, Phenomenon of Interest, Design, Evaluation, Research study) [18] (Table 1).

**Table 1:** SPIDER specifications

Dimension	Specification
Sample	Callers and operators in medical call centres in primary care, handling all kind of medical conditions
Phenomenon	Factors that affect the communication between caller and operator during telephone triage
Design	Not specified
Evaluation	All factors described affecting the communication
Research type	Qualitative, quantitative, and mixed-method studies

Original studies with qualitative, quantitative, and mixed-method design were included if (a) they described factors affecting communication between callers and operators in medical call centres, (b) the setting was medical call centres in primary care that managed all types of medical conditions from an unselected population, (c) they were published in English and (d) the full-text version was available.

### Search strategy and identification of studies

The search strategy was designed by three of the review group members (SLSF, VM, JV) and a research librarian (HW). The search was conducted in the databases MEDLINE (Ovid SP), Embase (Ovid SP), Cumulative Index to Nursing and Allied Health Literature (CINAHL) and Web of Science by the librarian on 15 April 2021. A search for new literature was conducted by another librarian (ISKS) on 26 June 2023, with the same search strategy (Additional file 2). In addition to the database search, the reference list of included studies was manually screened for supplementary literature.

### Selection of studies

Search results from the different databases were combined in an EndNote library file and uploaded to the systematic review management tool Covidence [19]. Duplicates were removed before two authors independently reviewed the titles and abstracts of the studies included. The authors voted for either inclusion or exclusion and were blinded to the other author’s vote during the screening process. When there was disagreement on inclusion/exclusion, an additional author reviewed the study and gave a third vote. The same strategy was used for the full-text screening. Each step of the screening process was conducted by the first author, SLSF, in collaboration with one or several of the co-authors, VM, JV and IHJ.

### Data collection, synthesis and quality assessment

Information about the first author, title, year of publication, country, and characteristics of the study (design, objective(s), sample size, study population characteristics, setting) was retrieved in Covidence by SLSF. A results-based convergent synthesis design was used to extract and synthesise the data [20]. The qualitative findings from the studies included were analysed using thematic synthesis, following three steps: free line-by-line coding, generation of descriptive themes and generation of interpretative/analytical themes [21]. The web-based programme EPPI reviewer [22] was used for free line-by-line coding. To create consensus on the extraction, the entire author group read a selection of studies and independently identified factors that were later discussed for agreement at joint meetings. SLSF then coded identified factors in all the studies and extracted all coded text from the EPPI reviewer into Excel files, where each theme was summarised and described.

The factors (analytical themes) identified in the qualitative studies constituted a framework for analysing the quantitative studies. The qualitative and quantitative results were presented individually and subsequently consolidated as an integration of findings. Throughout the process, the group of authors collaborated in discussions to overcome challenges and determine the next course of action.

Methodological limitations for each study were independently assessed and then discussed by SLSF and IHJ using the Mixed Methods Appraisal Tool (MMAT) [23].

## Results

**Fig. 1:**
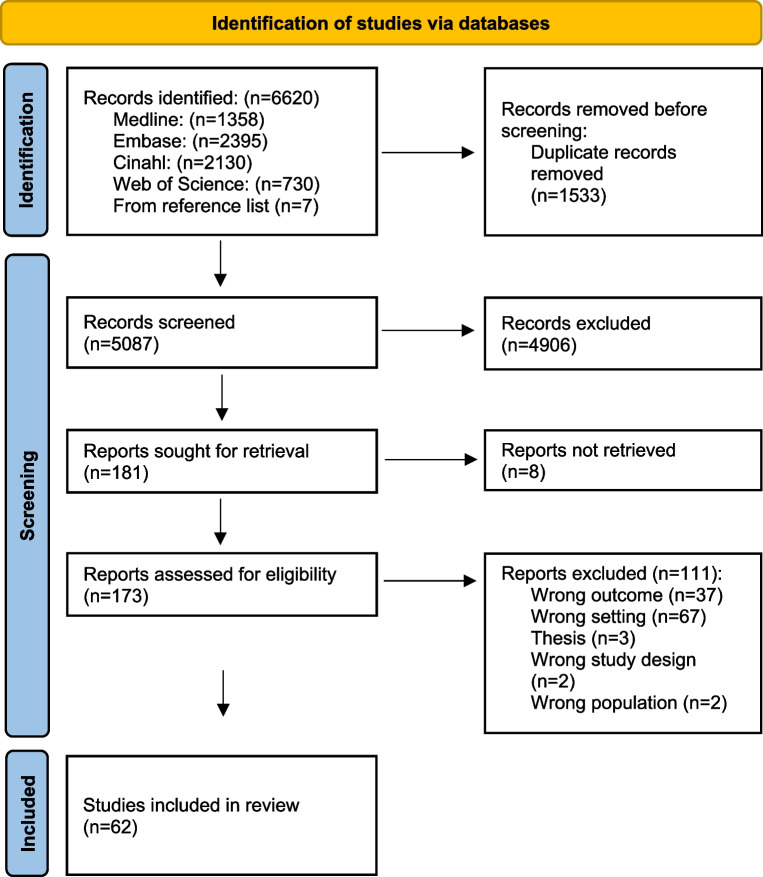
Prisma flowchart

**Fig. 2:**
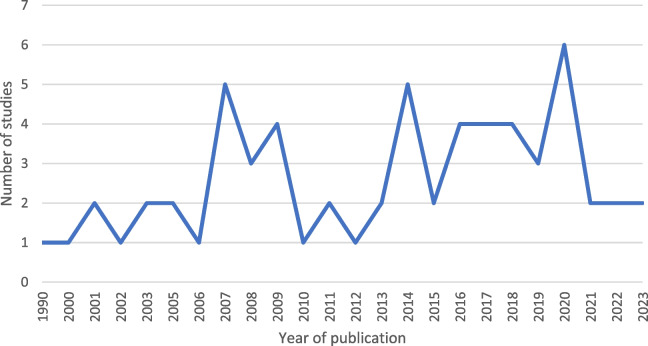
Year of publication of the studies (*N* = 62)

**Fig. 3:**
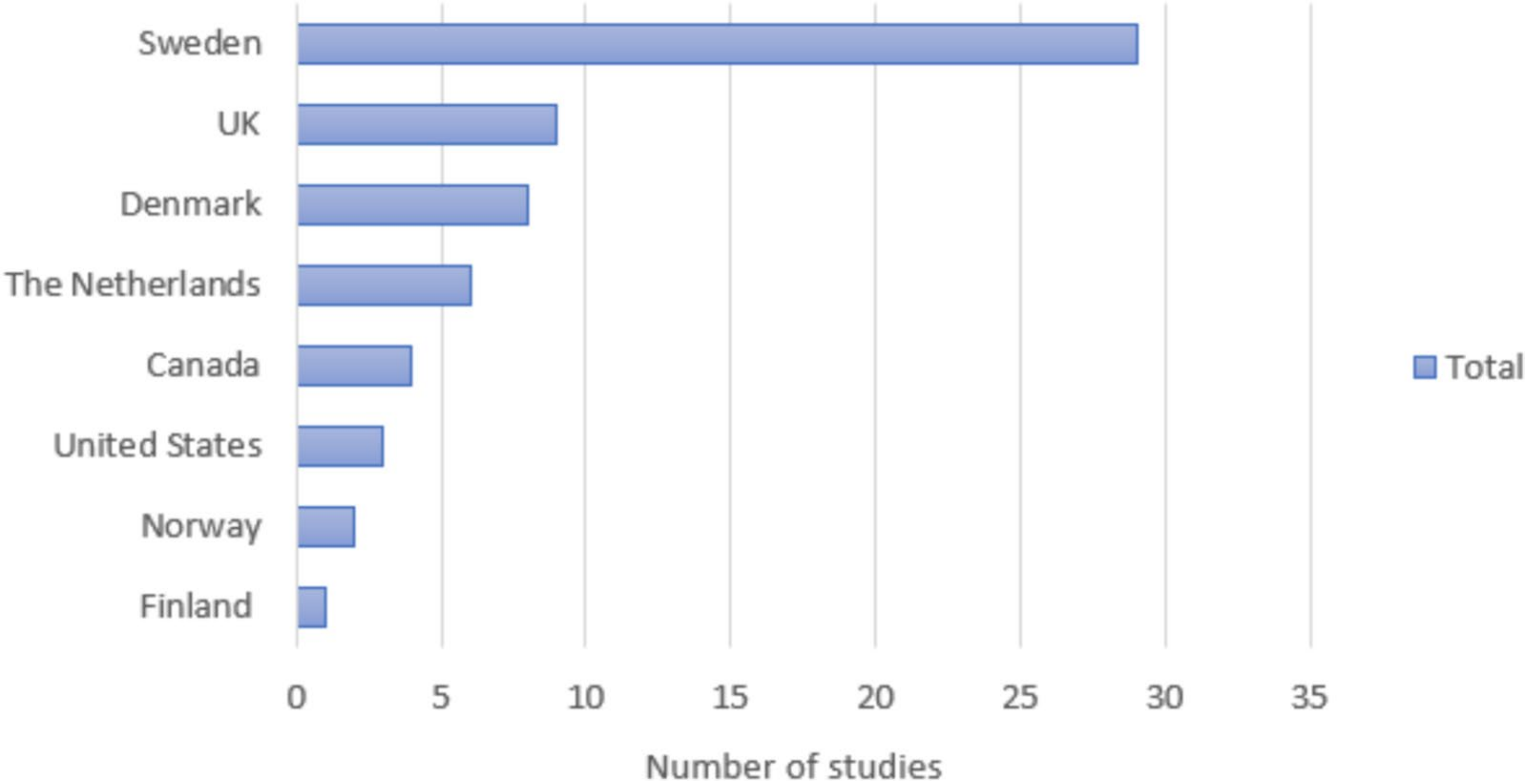
Country in which the studies (*N* = 62) were performed

The search yielded 6620 studies. After removing duplicates, 5087 titles and abstracts were screened. Only 173 studies were assessed in full text, and 62 studies were finally included in the review (Fig. 1). The 62 studies included comprised 40 qualitative studies, 16 quantitative studies, and six studies using mixed methods. Characteristics of each included study are available in Table 2. In the mixed methods studies, factors affecting communication were found in the quantitative part of one study [24], the qualitative part of three studies [25–27] and in both parts of two studies [28, 29]. The year of publication ranged from 1990 to 2023 (Fig. 2). The studies were performed in eight countries, with Sweden as the main contributor, accounting for 47% of the studies (Fig. 3). The primary data sources for the qualitative studies comprised interviews with either the operator or the caller. Additionally, open-ended survey questions and audio recordings of interactions between operators and callers were employed. In the case of quantitative studies, the predominant data sources were surveys and audio recordings. Out of all the studies, the operator’s view was explored in 28 studies, and the caller’s view in 18, while the remaining 16 studies explored both viewpoints. Altogether, 13 factors were identified and categorised into four main groups: organisational factors, factors related to the operator, factors related to the caller and factors in the interaction. Additional core elements describing each factor were also identified. An overview of the main themes, factors and core elements is presented in Table 3.

**Table 2:** Key information about the included studies (*N* = 62)

Author	Year	Country	Aim	Call centre setting	Study design and source of information	Perspective	Factors
**Qualitative studies**							
**Björkman**	2018	Sweden	Explore callers’ online communication about experiences and attitudes toward Swedish Healthcare Direct (1177)	Swedish Healthcare Direct (1177)	Content analysis of discussions on three internet-based forums, written by people with self-experienced situations (*n* = 230 pages)	Caller	**Organisational factors:** Availability **Factors related to the operator:** Knowledge and experience **Factors in the interaction:** Connection between operator and caller
**Björkman**	2018	Sweden	Describe telephone nurses’ experiences of encountering callers with mental illness	Swedish Healthcare Direct (1177)	Inductive content analysis of semi-structured interviews with nurses (*n* = 20)	Operator	**Organisational factors:** Availability, Working conditions **Factors related to the operator:** Knowledge and experience, Personal qualities, Communication strategies **Factors related to the caller:** Individual differences, Presented medical problem **Factors in the interaction:** Connection between operator and caller
**Cook**	2016	UK	Explore how mothers and grandmothers of young children experience a nurse led telephone-based healthcare service (NHS Direct), to uncover core factors which influence the level of satisfaction with the service	NHS Direct	Framework analysis of focus group interviews with callers (*n* = 17)	Caller	**Organisational factors:** Decision support system **Factors related to the operator:** Knowledge and experience **Factors in the interaction:** Faceless communication, connection between operator and caller
**Eriksson**	2019	Sweden	Describe telenurses’ experiences with difficult calls	Privat and public call centres and primary healthcare centres	Content analysis of semi-structured interviews with nurses (*n* = 19)	Operator	**Organisational factors:** Availability, Working conditions **Factors related to the operator:** Knowledge and experience, Personal qualities **Factors related to the caller:** Individual differences, Type of medical problem **Factors in the interaction:** Faceless communication, Connection between operator and caller, Third person caller, Communication barriers
**Eriksson**	2020	Sweden	Describe telenurses' strategies for managing difficult calls	Swedish Healthcare Direct (1177)	Content analysis of semi-structured interviews with nurses (*n* = 19)	Operator	**Organisational factors:** Availability, Working conditions, Decision support system **Factors related to the operator:** Knowledge and experience, Personal qualities, Communication strategies
**Erkelens**	2021	The Netherlands	Explore the interactional implications of either/or-questions on the interaction between people who call out-of-hours services in primary care and triage nurses who use a decision support tool called the ‘Netherlands Triage Standard’ during telephone triage	Out-of-hours services in primary care	Conversation analysis on audio-records (*n* = 68)	Caller and operator	**Factors related to the operator:** Communication strategies **Factors related to the caller:** Presented medical problem
**Ernesäter**	2009	Sweden	Describe telenurses’ experiences of working with computerised decision support systems and how such systems could influence their work	Swedish Healthcare Direct 1177	Content analysis of semi-structured interviews with nurses (*n* = 8)	Operator	**Organisational factors:** Availability, Working conditions, Decision support system **Factors related to the caller:** Individual differences
**Greenberg**	2009	United States	Develop a theoretical model of the process nurses use to deliver care over the telephone	Four telenursing services	Model constructing using semi-structured interviews with nurses (*n* = 10)	Operator	**Organisational factors:** Availability, Decision support system **Factors related to the operator:** Knowledge and experience, Communication strategies **Factors related to the caller:** Individual differences, Presented medical problem **Factors in the interaction:** Connection between operator and caller
**Gustafsson**	2020	Sweden	Describe patients’ experiences and perceptions of satisfaction with telephone nursing	Swedish Healthcare Direct 1177	Descriptive analysis of answers to open-ended survey questions answered by callers (*n* = 123)	Caller	**Organisational factors:** Availability **Factors related to the operator:** Communication strategies **Factors related to the caller:** Individual differences
**Hakimnia**	2014	Sweden	Explore the communication between telenurses and callers in authentic calls	Swedish Healthcare Direct 1177	Critical discourse analysis of audio-records (*n* = 20)	Caller and operator	**Factors related to the operator:** Communication strategies **Factors related to the caller:** Individual differences, Presented medical problem **Factors in the interaction:** Connection between operator and caller
**Holmström**	2002	Sweden	Describe how nurses experience the patient encounter when performing telephone advisory services	Primary Health Care Telephone Advisory Service	Semi-structured interviews with nurses (*n* = 5) analysed using the Empirical Phenomenological Psychological method	Operator	**Organisational factors:** Availability, Working conditions **Factors related to the operator:** Knowledge and experience, Communication strategies **Factors related to the caller:** Individual differences
**Holmström**	2007	Sweden	Explore the use of decision aid software programmes for telenursing, from the perspective of the users	Swedish medical call centre	Thematic analysis of semi-structured interviews with nurses (*n* = 12)	Operator	**Organisational factors:** Decision support system **Factors in the interaction:** Faceless communication
**Holmström**	2007	Sweden	Describe ethical dilemmas, in the form of conflicting values, norms and interests, which telenurses experience in their work	Swedish medical call centre	Thematic analysis of semi-structured interviews with nurses (*n* = 12)	Operator	**Factors related to the operator:** Personal qualities **Factors related to the caller:** Individual differences **Factors in the interaction:** Faceless communication, Third person caller
**Holmström**	2016	Sweden	Explore older persons` experiences of telephone advice nursing at primary healthcare centres	Swedish Healthcare Direct 1177	Content analysis of semi-structured interviews with callers > 65 years (*n* = 10)	Caller	**Organisational factors:** Availability **Factors related to the operator:** Knowledge and experience, Communication strategies **Factors related to the caller:** Individual differences **Factors in the interaction:** Faceless communication, Connection between operator and caller, Third person caller, Communication barriers
**Holmström**	2017	Sweden	Describe how telephone nurses define a frequent caller; and describe their experiences with calls from frequent callers to primary healthcare centres	Swedish Healthcare Direct 1177	Content analysis of semi-structured interviews with nurses (*n* = 10)	Operator	**Organisational factors:** Working conditions **Factors related to the operator:** Personal qualities **Factors related to the caller:** Individual differences **Factors in the interaction:** Connection between operator and caller
**Holmström**	2019	Sweden	Describe factors affecting the use of a decision support tool and experiences among telenurses in Swedish primary health care	Swedish primary healthcare centres	Content analysis of field observations (*n* = 32) and semi-structured interviews with nurses (*n* = 6)	Operator	**Organisational factors:** Working conditions, Decision support system **Factors related to the operator:** Knowledge and experience **Factors in the interaction:** Communication barriers
**Höglund**	2008	Sweden	Describe and explore gender aspects in telenursing as experienced by Swedish telenurses	Medical call-centre	Thematic analysis of semi-structured interviews with nurses (*n* = 12)	Operator	**Factors related to the caller:** Individual differences **Factors in the interaction:** Connection between operator and caller, Third person caller
**Jensen**	2022	Denmark	Explore the communication in telephone consultations between call-taker and callers describing back pain within 24 h before developing out-of-hours cardiac arrest	Copenhagen Emergency Medical Services (112 and 1813)	Content analysis of audio-records (*n* = 20)	Caller and operator	**Factors related to the operator:** Communication strategies **Factors related to the caller:** Presented medical problem
**Jensen**	2023	Denmark	Explore what characterised callers’ interpretation of experienced conditions where an approaching myocardial infarction was not initially recognised, and how the condition was described in the telephone consultation by the caller	Copenhagen Emergency Medical Services (112 and 1813)	Content analysis of audio-records (*n* = 28)	Caller and operator	**Factors related to the operator:** Communication strategies
**Kaminsky**	2013	Sweden	Explore and describe parents’ expectations and experiences of calling Swedish Healthcare Direct 1177 regarding paediatric health issues and discuss findings in the light of gender theory	Swedish Healthcare Direct 1177	Content analysis of semi-structured interviews with parents (*n* = 21)	Caller	**Organisational factors:** Availability **Factors related to the operator:** Knowledge and experience, Communication strategies **Factors related to the caller:** Individual differences **Factors in the interaction:** Third person caller, Communication barriers
**Leppänen**	2010	Sweden	Develop a framework for analysing how power operates in nurse–patient interaction and to empirically analyse power in the context of telephone-advice nursing in Swedish primary care	Telephone-advice nursing in primary care	Content analysis of audio-records (*n* = 276) and interviews with nurses (*n* = 18)	Caller and operator	**Factors related to the operator:** Knowledge and experience **Factors related to the caller:** Presented medical problem **Factors in the interaction:** Connection between operator and caller
**Lindberg**	2021	Norway	Explore how nurses assess callers with mild-to-moderate symptoms of respiratory tract infections and their views and experiences of triaging and counselling their callers	Local Emergency Medical Call Centre 116117	Systematic text condensation of four focus group interviews with nurses (*n* = 22)	Operator	**Organisational factors:** Availability, Decision support system **Factors related to the operator:** Personal qualities **Factors related to the caller:** Individual differences, Presented medical problem **Factors in the interaction:** Connection between operator and caller, Communication barriers
**Morgan**	2020	UK	Identify common points within the NHS111 call protocol where the resultant interactions appear vulnerable to misalignment. Explore the consequences of this misalignment for call outcome, specifically the clinical assessment and therefore the risk of system failure	NHS 111	Conversation analysis of audio-records (*n* = 40)	Caller and Operator	**Organisational factors:** Decision support system **Factors related to the operator:** Communication strategies **Factors related to the caller:** Presented medical problem
**Murdoch**	2014	UK	Compare doctors’ and nurses’ communication with patients in primary care telephone triage consultations	General Practice telephone triage	Conversation analysis of audio-records (*n* = 51) and video recordings on use of decision support systems (*n* = 10)	Operator	**Organisational factors:** Decision support system **Factors related to the operator:** Communication strategies
**Murdoch**	2015	UK	Understanding how nurses coordinate parallel activities of computer-based activity and talk with patients (or their proxies), focusing on how nurses deployed and integrated computerised decision support systems in the delivery of telephone triage for same-day appointments in primary care	General Practice Nurse-led telephone triage	Conversation analysis of audio-records (*n* = 22) and video recordings on use of decision support systems (*n* = 10)	Operator	**Organisational factors:** Working conditions, Decision support system **Factors related to the caller:** Individual differences
**O`Cathain**	2005	UK	Consider the extent to which NHS Direct facilitates patient empowerment in terms of helping people to be in control of their health and health care interactions	NHS Direct	Thematic analysis of semi-structured interviews with callers (*n* = 60)	Caller	**Organisational factors:** Availability, Decision support system **Factors related to the operator:** Communication strategies **Factors related to the caller:** Individual differences **Factors in the interaction:** Connection between operator and caller
**Pettinari**	2001	UK	Identify and describe nurses' perceptions of interactional practices they use to manage the absence of visual cues in telephone consultations with callers at an NHS Direct site	NHS Direct	Content analysis of semi-structured interviews with nurses and supervisors (*n* = 14 first interview, 12 second interview)	Operator	**Factors related to the operator:** Knowledge and experience, Communication strategies **Factors related to the caller:** Individual differences **Factors in the interaction:** Faceless communication
**Richards**	2007	UK	Explore users’ experiences of out-of-hours primary medical care	Out-of-hours primary care service	Thematic analysis of focus group and individual interviews with callers (*n* = 27)	Caller	**Organisational factors:** Decision support system **Factors related to the operator:** Communication strategies **Factors related to the caller:** Individual differences
**Röing**	2013	Sweden	Identify issues that could threaten patient safety in telenurses’ dialogues with callers	Swedish Healthcare Direct 1177	Content analysis of interviews based on stimulated recall sessions listening to real calls (*n* = 121) with nurses (*n* = 6)	Operator	**Organisational factors:** Availability, Working conditions **Factors related to the operator:** Knowledge and experience **Factors related to the caller:** Individual differences, Presented medical problem **Factors in the interaction:** Faceless communication, Communication barriers
**Röing**	2015	Sweden	Explore the direct experience of telenurses’ and call centre managers’ involvement in actual malpractice claims, with focus on factors that may have contributed to the claims and on the consequences of the claims	Swedish Healthcare Direct 1177	Content analysis of semi-structured interviews with nurses (*n* = 6) and managers (*n* = 5)	Operator	**Organisational factors:** Availability, Working conditions **Factors related to the operator:** Knowledge and experience, Communication strategies **Factors related to the caller:** Individual differences, Presented medical problem **Factors in the interaction:** Faceless communication, Communication barriers
**Skogevall**	2020	Sweden	Describe telephone nurses’ experiences of their encounters with frequent callers to Swedish Healthcare Direct	Swedish Healthcare Direct 1177	Content analysis of open-ended survey questions answered by nurses (*n* = 199)	Operator	**Organisational factors:** Working conditions **Factors related to the operator:** Knowledge and experience, Personal qualities **Factors related to the caller:** Individual differences **Factors in the interaction:** Connection between operator and caller
**Spek**	2023	The Netherlands	Better understand the interactional implication of discussing concerns during triage conversations between people who called for chest discomfort and triage nurses who use the Netherland Triage Standard tool	Out-of-hours services in primary care	Conversation analysis of audio-recorded calls (*n* = 68)	Caller and operator	**Organisational factors:** Decision support system **Factors related to the operator:** Communication strategies **Factors related to the caller:** Individual differences **Factors in the interaction:** Connection between operator and caller
**Ström**	2009	Sweden	Describe callers` perceptions of receiving advice via telephone helpline for medical care	Swedish medical care help line	Content analysis of semi-structured interviews with callers (*n* = 12)	Caller	**Factors related to the operator:** Knowledge and experience, Communication strategies **Factors in the interaction:** Connection between operator and caller
**Timpka**	1990	Sweden	First, develop a general description of telephone consultations in terms of the decision-making process and interpersonal communication. Second, analyse the dilemmas that receptionist nurses encounter	Health care centres	1) Content analysis of video recordings of telephone consultations using consultation mapping (*n* = 31). 2) Analysis of stimulated recall sessions using Habermas epistemological theory (*n* = 31)	Operator	**Factors related to the operator:** Knowledge and experience, Personal qualities **Factors in the interaction:** Faceless communication
**Tuden**	2015	Canada	Describe usability issues that emerged during a clinical simulation study of nurses working in a call centre	Simulated medical call centre	Content analysis of semi-structured interviews with nurses (*n* = 8) based on recall sessions using audio- and video-recordings of simulated calls (*n* = 16)	Operator	**Organisational factors:** Working conditions, Decision support system
**Wahlberg**	2001	Sweden	Describe callers' experiences of their contact with a medical call centre and to analyse the nature of their experience of consulting the nurses	Medical call centre regarding both emergency and urgent calls	Content analysis of open-ended survey questions answered by callers (*n* = 81)	Caller	**Organisational factors:** Availability **Factors related to the operator:** Communication strategies
**Wahlberg**	2005	Sweden	Explore what telephone nurses base their assessment on	Health-care call centre	Content analysis of interviews (*n* = 14) with nurses (*n* = 7) based on stimulated recall sessions	Operator	**Factors related to the operator:** Knowledge and experience
**Weir**	2008	UK	1) Consider the emotional work in encounters between nurse advisors and callers 2) Consider nurse advisors’ emotional experiences that pertain to wider issues relating to workplace and nursing management and the implementation of new ways of working	NHS Direct	Content analysis of in-depth interviews with nurses (*n* = 36), observations and field-notes	Operator	**Organisational factors:** Working conditions **Factors related to the operator:** Knowledge and experience, Personal qualities **Factors related to the caller:** Individual differences, Presented medical problem **Factors in the interaction:** Faceless communication
**Winneby**	2014	Sweden	Elucidate the care seeker’s situation and experiences of the care received after being triaged and directed to care centre on duty, although the telenurse in fact assessed their medical problems as corresponding to consultation with their regular doctor	Swedish Healthcare Direct 1177	Content analysis of semi-structured interviews with callers (*n* = 8)	Caller	**Organisational factors:** Availability **Factors related to the operator:** Communication strategies **Factors related to the caller:** Individual differences **Factors in the interaction:** Connection between operator and caller
**Yliluoma**	2020	Finland	Describe how telenurses experience interaction with callers	Call centre handling calls on behalf of the primary care health centre and district hospital	Content analysis of semi-structured interviews with nurses (n = 9)	Operator	**Organisational factors:** Availability, Working conditions **Factors related to the operator:** Knowledge and experience, Personal qualities, Communication strategies **Factors related to the caller:** Individual differences, Presented medical problem **Factors in the interaction:** Faceless communication, Connection between operator and caller, Third person caller, Communication barriers
**Quantitative studies**							
**Allan**	2014	UK	Investigate whether: (1) stress predicts cognitive failures in telephone nurses; (2) stress affects the speed and accuracy of nurses’ information processing; and (3) any such changes in cognitive efficiency are related to changes in the decisions that nurses make	Scottish health helpline NHS-24	Descriptive study on measures of stress, cognitive performance, and work performance in nurses (*n* = 152)	Operator	**Organisational factors:** Availability
**Boutin**	2006	Canada	Evaluate the effects of a continuing education activity, based on cognitive and andragogic approaches, on the quality of the intervention by Info-Sant ´e CLSC nurses dealing with asthmatic patients and on the number of referrals to acute and emergency centres	Info-Santè CLSC	Prospective intervention study using audio-records: Pre-training (*n* = 24 nurses/44 calls). After 3 months (*n* = 25 nurses/48 calls). After 9 months (*n* = 23 nurses/42 calls)	Operator	**Organisational factors:** Knowledge and experience
**Derkx**	2009	The Netherlands	Assess the quality of communication skills of operators, working at out-of-hours centres, and to determine the correlation between the communication score and the duration of the telephone consultation	Out-of-hours centre	Descriptive study using Roter Interaction Analysis System (RIAS) communication score to assess audio-records of simulated calls (*n* = 357)	Caller and operator	**Organisational factors:** Availability
**Ernesäter**	2014	Sweden	Compare communication patterns in calls subjected to a malpractice claim with matched controls	Swedish Healthcare Direct 1177	Case–control study using Roter Interaction Analysis System (RIAS) to assess audio-records (*n* = 26 cases/ 26 controls)	Caller and operator	**Organisational factors:** Availability **Factors related to the operator:** Communication strategies
**Giesen**	2007	The Netherlands	Explore the association between negative patient evaluation of nurse telephone consultations and characteristics of patients and general practitioner cooperatives	General practitioner cooperative	Descriptive study using postal patient questionnaires (*n* = 2583)	Caller	**Factors related to the caller:** Individual differences
**Giesen**	2008	The Netherlands	Explore the incidence rates of rude or aggressive patient behaviour in general practitioner out-of-hours care and to explore factors associated with such behaviour	General practitioner cooperatives	Descriptive study using medical records (*n* = 36,259)	Caller	**Factors related to the caller:** Presented medical problem
**Graversen**	2020	Denmark	Compare the quality of communication in out-of-hours telephone triage conducted by general practitioners, nurses using a computerised decision support system and physicians with different medical specialties, and to explore the association between communication quality and efficiency, length of call and the accuracy of telephone triage	General practitioner cooperative and medical helpline 1813	Natural quasi-experimental study using the tool Assessment of quality in telephone triage (AQTT) to analyse audio-records of calls handled by general practitioners (*n* = 423), nurses (*n* = 430) and physicians (*n* = 441)	Operator	**Organisational factors:** Decision support system
**Gustafsson**	2016	Sweden	Explore the influence of nurse-led self-care advice on healthcare utilisation and patients’ satisfaction with telephone nursing	Swedish Healthcare Direct 1177	Descriptive study using questionnaires answered by callers (*n* = 285)	Caller	**Organisational factors:** Availability **Factors related to the operator:** Communication strategies
**Hagan**	2000	Canada	Address perceived accessibility, satisfaction with care, and the development and use of self-care abilities due to nursing interventions	Info-Santé Local Community Service Center (CLSC)	Descriptive study using questionnaires answered by callers (n = 4696)	Caller	**Factors related to the caller:** Individual differences
**Hansen**	2011	Norway	Investigate how callers understand the information given by telephone by registered nurses in a casualty clinic, to what degree the advice was followed, and the final outcome of the condition for the patients	Local emergency medical call centre	Descriptive study using audio-records of calls and structured interviews with the callers (*n* = 100) where callers had received medical advice by nurse as a sole response	Caller	**Factors related to the caller:** Presented medical problem
**Huibers**	2012	The Netherlands	To explore the impact of quality of consultation and estimated urgency on the appropriateness of decisions	General practitioner cooperatives	Descriptive study using a quality measurement instrument (HAAKplus) to analyse audio-records (*n* = 6739)	Operator	**Factors related to the caller:** Presented medical problem
**Leclerc**	2003	Canada	Validate users’ perception of nurses’ recommendations to look for another health resource among clients seeking telephone advice. To analyse the effects of different users’ and call characteristics on the incorrectness of the self-report	Info-Sante ´CLSC	Descriptive study using questionnaires answered by callers (*n* = 4696)	Caller	**Factors related to the caller:** Presented medical problem
**Moscato**	2007	United States	Examine predictors of patient satisfaction with telephone nursing services	Telephone advice services	Descriptive study using a nurse questionnaire (*n* = 12), audio-recorded calls, call logs and callers questionnaires (*n* = 1939). Each call was also listened to and coded using a 50-item call description tool together with an interpersonal communication style index	Caller and operator	**Organisational factors:** Knowledge and experience, Communication strategies
**Njeru**	2017	United States	Determine the utilisation characteristics of a primary care triage call centre by patients who require interpreter services	Primary care triage call centre	Cohort study using register data and patient electronic health record to obtain patient demographics and call characteristics to compare callers with limited English proficiency (*n* = 587) to English proficient (587) callers	Caller	**Factors in the interaction:** Communication barriers
**Ström**	2011	Sweden	Describe how patients’ sex, age, education level and care level influenced their perceptions of care encounters with the medical care help line	Medical Care Help Line	Descriptive study using questionnaires answered by callers (*n* = 517)	Caller	**Factors related to the caller:** Individual differences
**Vilstrup**	2019	Denmark	Compare communicative parameters in general practitioner-led and nurse-led out-of-hours telephone triage and to discuss differences in relation to patient-centred communication and safety issues	General practitioner cooperative and Medical Helpline 1813	Observational study comparing audio-recorded general practitioner-led (*n* = 100) to nurse-led (*n* = 100) telephone triage calls	Caller and operator	**Organisational factors:** Decision support system
**Mixed-Method studies**							
**Ernesäter**	2016	Sweden	Describe telephone nurses' and callers' communication, investigate relationships within the dyad and explore telephone nurses' direct response to callers' expressions of concern	Swedish Healthcare Direct 1177	Descriptive quantitative analysis using Roter Interaction Analysis System and qualitative content analysis of calls (*n* = 25)	Caller and operator	**Factors related to the operator:** Communication strategies
**Gamst-Jensen**	2017	Denmark	Describe situations of under-triage in context, to assess the quality of under-triaged calls, and to identify communication patterns contributing to under-triage	Medical helpline 1813	Descriptive quantitative analysis and qualitative thematic analysis of calls (*n* = 327)	Caller and operator	**Factors related to the operator:** Personal qualities, Communication strategies **Factors related to the caller:** Individual differences, Presented medical problem **Factors in the interaction:** Connection between operator and caller, Third person caller, Communication barriers
**Gamst-Jensen**	2018	Denmark	Explore the ability of callers to quantify their degree of worry, the association between their degree of worry and variables related to the caller, the effect of the degree of worry on triage outcome, and the thematic content of the caller’s worry	Medical helpline 1813	Simultaneous convergent design combining descriptive statistics and thematic analysis of calls (*n* = 180)	Caller	**Factors related to the operator:** Communication strategies
**Gren**	2022	Denmark	Investigate 1) How video triage versus telephone triage in children was experienced by parents and call-handlers, and 2) call-handlers` evaluation of the video triage projects	Medical helpline 1813	Thematic analysis, content analysis and descriptive analysis using questionnaires and semi-structured interviews / Parents’ questionnaires (*n* = 567 study 1 and *n* = 168 in study 2), Operator questionnaires (*n* = 1245), Operator interviews (*n* = 7)	Caller and operator	**Organisational factors:** Working conditions **Factors related to the operator:** Communication strategies **Factors in the interaction:** Third person caller, Communication barriers
**Thilsted**	2018	Denmark	Examine the relation between patients’ illness representations, presented in telephone consultation to out-of-hours (OOH) services, and self-reported degree-of-worry (DOW), as a measure of self-evaluated urgency	Medical helpline 1813	A convergent parallel mixed-method design using Degree-Of-Worry scale for quantitative data and thematic analysis of audio-recorded calls for quantitative data. Common-Sense Model of Self-Regulation were used as framework	Caller	**Factors related to the caller:** Individual differences, Presented medical problem
**Wahlberg**	2003	Sweden	Identify problems, difficulties and disadvantages that telephone nurses with varying degrees of experience had met during their professional careers	Health-care call centre	Content analysis of open-ended survey questions with nurses (*n* = 25)	Operator	**Organisational factors:** Availability, Working conditions **Factors related to the operator:** Knowledge and experience, Personal qualities, Communication strategies **Factors related to the caller:** Individual differences, Presented medical problem **Factors in the interaction:** Faceless communication, Connection between operator and caller, Third person caller, Communication barriers

**Table 3:** Overview of factors and core elements belonging to each main theme

Main theme	Factors	Core elements
**Organisational factors**	**Availability**	Accessibility
		Operator resources
		Availability of other resources
	**Working conditions**	Construction of the workspace
		Organisational policies and attitudes
		Technical aids
	**Decision support system**	Ensuring quality
		Structuring the conversation
**Factors related to the operator**	**Knowledge and experience**	Medical and organisational knowledge
		Personal and clinical experience
		Training and education
	**Personal qualities**	The operators’ appearance
		Control over own emotional reactions
	**Communication strategies**	Active use of tone and rhythm
		Take control and structure the communication
		Give information, instructions, reassurance and confirmation
		The design of questions
		Assessment techniques
**Factors related to the caller**	**Individual differences**	Sociodemographic factors
		Influence of drugs and alcohol
		Callers’ attitude
		Callers’ expectations
		Emotional stress
		Callers’ level of knowledge and experience
	**Presented medical problem**	Callers’ choice of words
		Urgency level
		Mental illness
**Factors in the interaction**	**Faceless communication**	Dependent on the caller’s description
		Anonymity
	**Connection between operator and caller**	A positive relation
		Pre-established relationship
		A power asymmetry
	**Third-person caller**	Second-hand information
		Callers’ proximity to the patient
		Confidentiality requirements
	**Communication barriers**	Distractions in the environment
		Language barriers

### Qualitative findings

#### Organisational factors

The organisational theme contained three factors: availability, working conditions, and decision support systems.

##### Availability

The availability of the service affected the communication. Queuing and waiting to get through to the operator were described by both callers and operators as negatively affecting communication [27, 30–33]. Callers found the uncertainty of the waiting time frustrating, which could make them irritated and angry, requiring operators to spend extra time to calm them down [27, 30–32].

When operator resources did not correspond to needs, the queues of callers increased and the operators reported a high level of stress [9, 27, 31, 32, 34–36]. The operators communicated faster and more mechanically, which could lead to quick decisions being made based on little information [27, 31, 32, 34–38]. Time to express their needs was highlighted as important for the callers [39, 40]. Sufficient operator resources also allowed for collaboration with other colleagues to discuss difficult issues [8, 9, 27, 35], which increased operators’ self-confidence [41] and callers’ faith in the advice given [39, 42, 43].

The operators’ ability to make decisions during the conversation was affected by shortages of other resources, such as doctors on call, ambulances and mental health services [9, 27, 31, 32, 34, 37, 44, 45]. When a shortage of resources, the operators had to spend time on explaining the lack of available resources [31, 32].

##### Working conditions

The construction of the workspace was described as influencing communication [31, 41]. The opportunity to move and stretch helped the operators to concentrate better [31], and proximity between the operators’ workspaces allowed for collaboration [41].

Organisational attitudes affected how rigidly the operators used guidelines [31, 35, 46–48]. If the employer had high-efficiency expectations, the operators felt they were monitored and described the same effect as we found under lack of operator resources: less time for good communication [9, 32, 34, 45]. The organisation of work shifts could reduce the quality of communication, due to physical and psychological limitations during long shifts or night shifts [35, 37, 47].

Technology, such as the use of video, could clarify or prevent misunderstandings [28]. However, multitasking between different technical aids while gathering and interpreting information from the callers was described as cognitively demanding [35, 48, 49]. Beginners spent most of their cognitive capacity on technology, which suppressed the use of their own medical knowledge and communication skills [35]. Technical failure caused problems with focusing, stress and a lack of control, which led to disruptions in the conversation [27, 31, 34, 45, 50, 51].

##### Decision support systems

Decision support systems (DSS) were described by the operators as ensuring quality by giving structure to the conversations, providing evidence-based knowledge, and being a checklist for important questions when the operators’ own clinical knowledge and experience were limited [8, 41, 48, 50, 52]. Also, callers were more receptive to advice when informed that it came from the DSS [36, 48, 50].

However, DSS structured the conversation in a way that deviated from normal conversations, particularly when operators followed a DSS with a fixed checklist structure [49, 53, 54] that used standardised sentences and closed yes/no questions [54, 55]. Consequently, this restricted the caller’s provision of a detailed description of the situation and made the operators convert all reasons for calling into a problem that suited the system [54]. The operators described feeling controlled, directed, passive and less attentive to the callers when using a fixed checklist structure [50, 56]. The callers described the operators’ use of checklists as frustrating and impersonal, due to an increased number of questions that sometimes seemed irrelevant [38, 57, 58]. If the DSS lacked information, was non-intuitive or used medical terminology, the operators spent time searching the tool or translating words into everyday language, which led to pauses in the conversation [49–51]. In contrast, the operators’ attention was notably more oriented to the ongoing conversation when they did not use DSS [53].

#### Factors related to the operator

Factors related to the operator comprised three categories: knowledge and experience, personal qualities, and communication strategies.

##### Knowledge and experience

Sufficient medical knowledge was described by the operators as essential for asking the right questions and being confident when gathering information and making decisions [32, 47]. Similarly, the operators’ organisational knowledge was described as a basis for decisions and thereby also the information conveyed to the callers [8, 9]. Callers described greater trust when they spoke to operators who were more competent than themselves [7, 33, 39, 42].

During calls, operators used personal and professional experience to assess symptoms and problems. This influenced the questions asked, the operators’ perception of the situation and the advice given [34]. As they became more experienced, operators developed tacit knowledge and the ability to visualise the patient’s situation. Tacit knowledge was explained as intuition or a gut feeling, which made the operators able to read between the lines and catch information that was not verbalised [8, 27, 31, 37, 58–60]. Visualisation was described as crafting a mental image of the patient’s circumstances and served as a means of promoting understanding [31, 44].

Training and education affected communication by increasing operators’ competence in, e.g. communication strategies and medical knowledge, which enhanced the operators’ overall performance and sense of security [8, 9, 27, 31, 35, 41, 46, 48].

##### Personal qualities

The callers reported that the operators’ appearance in the conversation influenced their experience of the call. The operators’ positive attitude was important for the callers, as they felt vulnerable when calling the service [30, 40, 42, 58]. A positive attitude was also emphasised as important for achieving good communication [26, 31, 44–46]. If the callers experienced the operators as dismissive, unfriendly, arrogant or disrespectful, this could lead to communication characterised by anger, irritation and mistrust [7, 33, 39, 42]. Both operators and callers described a correlation between confident operators and feeling reassured [30, 36, 40].

In response to callers’ emotional state (anger, indignation, sadness) or situation (death, abuse), the operators could become emotionally affected [9, 31, 32, 47], which made the conversation quite demanding, especially if the caller was aggressive. The operators described that having control over their reactions during the calls was crucial to achieve effective and good communication with the caller [7, 27, 31, 40, 41, 47].

##### Communication strategies

Different articulation (tone and rhythm) was used actively to create a calm atmosphere (speaking calmly), emphasise important information (articulating clearly), calm an aggressive caller (speaking calmly and in a deeper voice) and show that one has understood the seriousness of the problem (speaking faster) [8, 31, 40, 41, 59].

Taking control and structuring the communication helped the operator maintain the direction of the conversation and choose what to investigate further [8, 31, 56]. The necessary control and structure had to be balanced against giving the caller enough time to explain the situation [31, 42, 59]. Listening actively and communicating in a calm, empathetic and attentive way helped the operator grasp the situation and gain the caller’s trust [8, 9, 38, 40, 41, 54, 59]. It was a pitfall if the operator had interpreted the patient’s symptoms in one direction, and worked to confirm that direction, thereby overlooking important information from the caller [61]. Pausing the conversation was a helpful strategy if the conversation got out of control emotionally [31, 37, 41].

Providing information, instructions, reassurance, and confirmation were described by operators and callers as beneficial communication strategies [7, 31, 37–39, 41–43, 59] that could calm the callers [30, 37, 41]. Callers emphasised the need to understand the reasoning behind the operators’ questions and decisions, and they also wanted to be informed about the causes of symptoms and how to deal with them [38–40, 57]. By summarising the conversation and allowing the callers to participate in joint decision-making, operators facilitated a common understanding of the situation and a feeling of security [7, 8, 38, 39, 43, 61]. The use of common language and tailored advice created a shared understanding of the content of the conversation and enabled the caller to apply the advice given [26, 28, 31, 37, 39, 41, 42]. Operators who did not expect a highly urgent situation allowed callers to speak freely to a greater extent than if an urgent situation was expected [62].

The design of the questions affected the quality of the information collected. Asking questions in the present tense (“How can I help you now?”) helped the callers focus on the here and now [41]. Operators described that open-ended questions gave a broader overview of the patients’ situation, compared to closed questions [9, 35]. Yet using closed questions seemed to be a good strategy to clarify specific elements [53, 54, 63]. However, simple questions based on yes/no and either/or could be challenging to answer if they did not suit the situation [54, 63]. Callers tended to only answer the last question if the operators asked two or more questions immediately after each other [55].

Exploring the caller’s concern was described as a good strategy to obtain additional information [25, 55]. Furthermore, assessment techniques such as listening for respiratory sounds and instructing a third person to perform physical examinations were described as useful [42, 59].

#### Factors related to the caller

The factors related to the caller were categorised into two groups: individual differences and the presented medical problems.

##### Individual differences

Sociodemographic factors, such as gender, age, culture, level of education, and place of residence, affected communication [27, 31, 57, 64, 65]. Different ways of describing illness and expressing needs between cultures made it difficult for callers and operators to understand each other [27, 64, 65]. Age, gender, and place of residence could affect how symptoms were presented and how actively the caller participated in finding solutions. Older people, people living in rural areas, and men tended to neglect or underreport serious symptoms [31, 64], and the operators had to take this into account.

If the callers were under the influence of drugs or alcohol, the conversations could become more aggressive and challenging due to how the drugs affected the callers’ ability to talk, describe their problems and listen [9, 26, 27, 46].

The callers’ attitude toward the system and the operators was described as affecting the tone of the conversation [8, 50, 59]. Callers described how experience from previous encounters affected their trust in the operators and their decisions [39, 40]. Callers’ expectations of the service did not always match what the healthcare system should or could offer, which could make it challenging to reach an agreement [27, 32, 35, 36, 50]. This was particularly challenging if the caller had decided on the outcome in advance and was not receptive to the operator’s assessment [27, 32, 36]. Callers described how they exaggerated their symptoms or the situation if they had to legitimise the contact [43].

Emotional stress and callers’ ability to control their emotions affected how the conversation developed. Diminished control hindered rational thinking, augmented problems with listening and taking in what was said and made it challenging to describe the symptoms and adhere to the operator’s instructions [9, 26, 27, 32, 34, 37, 38].

The callers’ level of knowledge and experience influenced communication. Operators described how the conversation became more challenging when callers lacked knowledge about illness, normal bodily functions, and the organisation of the health services [27, 47, 56]. The knowledge gap acted as a barrier to shared understanding, as callers then struggled to describe their situation, held back information due to uncertainty or responded without comprehending the operators’ questions [49, 55, 56]. A disparity in situational awareness between operators and callers could arise when the callers had conducted online searches prior to their call and misinterpreted the information [34, 43, 45].

##### Presented medical problem

Callers’ interpretations of their symptoms were based on knowledge and previous experience and affected their choice of words when describing symptoms [29, 61, 66]. When callers were uncertain of which symptom or observation that was important, or lacked knowledge, terminology, or ability to succinctly describe the symptom, they often presented more information than necessary, or gave a vague or wrong description [31, 32, 34, 54, 56, 63]. Given their experience with callers who could both exaggerate and downplay their symptoms, operators described an underlying skepticism toward callers’ descriptions that complicated interpretation of the symptoms [27, 32, 36]. Another obstacle described was when callers themselves presented a diagnosis or solution, and the operators chose to draw conclusions based solely on this information [35].

The level of urgency of the medical situation affected the communication. A conversation with high acuity was characterised as more streamlined, straightforward, and easy to handle, due to the need for measures to be initiated quickly [8].

Operators described calls regarding mental illness as different from calls regarding somatic illness [8, 9, 27, 47]. Due to shame, callers often hid the true reason for their call behind diffuse symptoms [9]. Conversations dealing with mental illness were described as difficult, time-consuming, requiring multiple nursing skills, exhausting, and emotionally demanding for the operators, which made the operators more reluctant towards these conversations [8, 9, 27, 47].

#### Factors in the interaction

The following factors were related to the interaction: faceless communication, connection between operator and caller, third-person caller and communication barriers.

##### Faceless communication

Operators described themselves as completely dependent on the caller’s description, which made it difficult to create a correct picture of the symptoms and situations, especially when describing skin symptoms or characteristics of children’s symptoms [27, 31, 32, 34, 35, 44, 59, 65]. Not being able to read or use body language made it more difficult to clarify words, read expressions and interpret and monitor responses to the information given, compared to face-to-face conversations [27, 31, 32, 39, 59, 65]. Simultaneously, a lack of visual clues was described as preventing premature judgments based on visual impressions alone [59].

The anonymity of the faceless conversation had advantages and disadvantages. It could make it easier to discuss embarrassing topics [47, 58, 59]. The operators also described it as positive that they could express emotions through body language with no effect on the caller [31]. However, operators described how uncertainty regarding caller identity limited information sharing [59, 65].

##### Connection between operator and caller

A positive relation between the caller and operator facilitated seamless communication, which made it more likely that the operator would gain a comprehensive understanding of the caller’s situation [8]. Similarity between caller and operator (e.g. the same gender) was described as a factor that eased the connection [64]. The operators described how sympathy came more naturally when they could recognise themselves in the situation [32]. Feeling sympathy for the caller’s situation made the operators more engaged in the conversation, in contrast to calls without a sympathetic approach, where the communication became more direct and technical [56].

A pre-established relationship between the caller and the operator promoted continuity, insight, and trust, and increased the possibility of finding personalised solutions [9, 39, 46]. Previous knowledge could also be a disadvantage, as in the case of frequent callers, who call many times about the same problems. For this group, the operators described the conversations as stressful, frustrating, time-consuming, less empathetic and with a different structure compared to other calls, with less listening and less use of decision-making support [27, 45, 46].

For the callers, having to repeat information created frustration [43, 58]. Information from previous contacts in the patient’s medical records could illuminate the situation, help the operator build on previous conclusions and prevent the caller from repeating information [8, 31, 32, 36, 45].

A power asymmetry in the interaction was described, where the operators, as the professionals and the “door openers’” to medical help, had the most power [26, 38, 55, 56, 66]. Yet, callers described themselves as personal experts and emphasised the importance of not being devalued and considered solely as a source of information [7, 38, 39, 43]. A power struggle could affect the communication [26, 33, 38, 43]. To equalise the balance of power, callers used methods such as expressing concern to elicit empathy, arguing until they achieved the desired action, and presenting the problem as an order or as a recommendation from someone with greater authority [66].

##### Third-person caller

Second-hand information from a third-person caller (not the patient) was described as difficult to trust and interpret and required more questions to be asked [27, 28, 31, 32, 64]. Parents expressed how it was challenging to interpret and describe children’s symptoms objectively and that feelings and fear influenced how the information was provided [42]. Nevertheless, talking to a third person provided information of great importance when the patient was prevented from speaking for themselves [31, 39]. The third person’s proximity to the patient, in physical or relational distance, affected the quality of the information given [26, 64].

Confidentiality requirements hindered the flow of information between third-person callers and the operators, as the operators had to be careful not to share specific details with a third person [31]. Talking about a patient without the patient’s consent was considered an ethical dilemma, where the operator had to deal with safeguarding both the patient’s and the caller’s autonomy [65].

##### Communication barriers

Distractions in the caller’s or operator’s environment made it difficult to hear the other party and took focus away from the conversation. As a result, it became uncertain whether the caller or operator was listening properly or understanding the content of the conversation [31, 35, 39].

Language barriers were described as creating uncertainty and misunderstandings, delaying and complicating the progression of the conversation and making it more problematic to use decision support systems [27, 28, 32, 34, 36, 42, 48, 67]. Similar effects were also observed in calls where the caller had impaired hearing or speech [26, 31, 39].

### Quantitative findings

#### Organisational factors

Quantitative studies had results that shed further light on all three organisational factors: availability, working conditions, and decision support system. The results from a survey of callers’ satisfaction showed a negative correlation between perceived unreasonable waiting time before the call was answered and overall satisfaction with the service [68]. An observational study exploring the cognitive impact of stress in operators found that higher stress levels increased the number of cognitive failures and affected the operator’s decisions, but also made the operators process information more quickly [69].

A study that assessed the quality of communication using a list of assessment items (RICE communication list) found a positive correlation between time spent per call and quality of communication [15]. However, a study comparing calls ending in malpractice claims with other calls found no significant difference in time spent per call [70].

A survey investigating how the use of video was experienced by both operators and callers showed that the integration of video was considered to enhance safety and elevate the overall quality of communication [28].

Two observational studies using audio records to compare calls handled by nurses utilising DSS and doctors not utilising them observed that nurses tended to ask more questions, often adopting a checklist-style approach [5, 71]. When communication quality was compared between the two groups using a validated quality assessment tool (AQTT—Assessment of Quality in Telephone Triage), there was no significant difference in the overall quality [5].

#### Factors related to the operator

In relation to the operator, the factors of knowledge and experience and communication strategies were supported by quantitative studies. Personal qualities were only described in qualitative studies.

A study examined predictors of callers’ satisfaction by matching the content of audio-recorded calls with caller questionnaire data. They found that expectations fulfilled by the operator in terms of listening, clarity, cooperation and perceived competence were strong predictors of caller satisfaction [72]. An intervention study examining the effect of educational activities on the quality of the information provided during telephone triage found a positive impact on quality in the short term, but the effect did not persist over time [73].

A study comparing calls that resulted in reported medical errors with other calls found that active listening and checking a shared understanding were less present in calls that resulted in reported medical errors [70]. This same study also showed that when operators used more open-ended questions, callers provided significantly more medical information than in calls with fewer open-ended questions. Another study examining callers’ satisfaction with receiving self-care advice found that callers’ satisfaction with the help received was dependent on whether the caller felt reassured after the call [68].

#### Factors related to the caller

Evidence from quantitative studies elaborated on both factors related to the caller: individual differences and presented medical problem. A user satisfaction survey showed that people with a lower level of education were less satisfied with the information and advice given, due to problems with understanding the content [74]. Another survey study found that mismatch of expectations was associated with low satisfaction with the call [75]. Callers with a high degree of concern needed more reassurance due to worries about negative consequences of their illness [29].

An observational study using a communication quality measurement tool showed a negative association between urgency level and the quality of communication [6]. Another study found that very urgent calls were characterised by less rude and aggressive behaviour than less urgent calls [76].

Observational studies found that calls regarding mental illness lasted longer than the other calls [77] and had a greater risk of misunderstandings [78] and that callers with mental health problems were at greater risk of being rude and aggressive when talking to healthcare professionals than other callers [76].

#### Factors in the interaction

The only interactional factor supported by quantitative results was communication barriers.

Two observational studies comparing non-fluent speakers to fluent speakers found that trust and satisfaction were lower in non-fluent than in fluent speakers [67, 77]. One of the studies found that communication time was on average longer for the non-fluent speakers [67].

### Integration of quantitative and qualitative findings

**Fig. 4:**
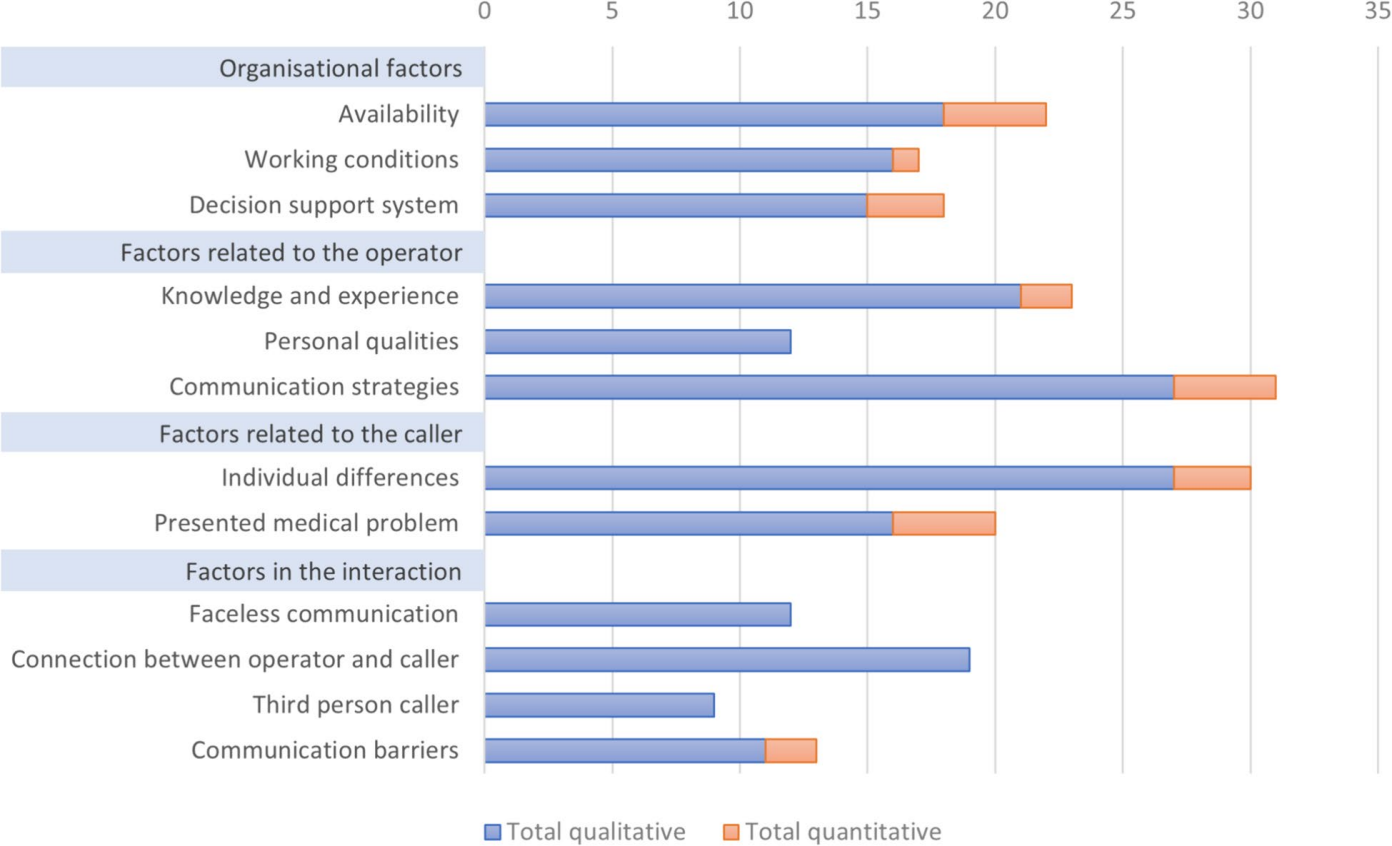
Number of studies (*N* = 62) supporting each factor

An overview of the factors and where the quantitative findings either expand upon or corroborate the established qualitative framework is presented in Fig. 4. In addition, a more comprehensive overview of the factors distributed over each individual study can be found in Additional file 3. The assessed quality of the studies was generally high, and 48 of 62 studies were found to have the maximum score. The results of the assessment are further described in Additional file 4.

#### Organisational factors

The number of studies and the diversity in terms of population, country and perspectives studied suggested that availability [8, 9, 15, 27, 30–43, 50, 68–70] strongly affected communication. Resource shortages affected accessibility of the service, operators’ stress and callers’ satisfaction negatively. Time pressure and stressful working environment made the operators rush through the communication to the extent that it could become a patient safety problem. Having enough time to gain a comprehensive overview of the patient’s situation seemed essential for quality. However, divergent findings when examining the link between the duration of the conversation and the quality of the communication imply that time alone may not serve as a reliable indicator of conversation quality [15, 70].

Decision support systems were also well-documented in both qualitative and quantitative studies [5, 8, 36, 38, 41, 48–55, 57, 58, 71]. The DSS’s role in structuring the conversation was described particularly thoroughly [5, 38, 48–58, 71]. If the operators adhered strictly to the tool’s algorithms during the information collection, the conversation was more strained and did not develop naturally. Also, the use of DSS increased the number of questions asked and the frequency of checklist-style questions used. Nevertheless, quantitative data did not reveal any difference in the overall quality of the conversation when using DSS compared to not using DSS [5].

The factor working conditions was mainly supported by qualitative studies [9, 27, 31, 32, 34, 35, 37, 41, 45–51]. One mixed methods study from Denmark examined the effect of video as a technical aid [28]. This study examined both callers’ and operators’ perspectives and found that video reassured both parties by contributing to an expanded understanding of the situation. However, the quality of this study was compromised due to the substandard description of some of the methods and data sources mentioned in the paper.

#### Factors related to the operator

Knowledge and experience [7–9, 27, 31–35, 37, 39, 41, 42, 44, 46–48, 58–60, 72, 73] were supported by both qualitative and quantitative findings. The integration indicated that the operators’ medical and communication skills affected the information obtained. These skills also emerged as strong predictors for establishing trust and confidence between the two parties during communication. Training and education were described by the operators as improving quality, which was also quantitatively supported by one study [8, 9, 27, 31, 35, 41, 46, 48, 73]. However, the effect seemed to decrease over time, suggesting that training needs to be repeated [73].

The number and diversity of studies [7–9, 24–28, 30, 31, 35, 37, 38, 40–43, 53–57, 59, 61–63, 68, 70, 72] supported that operators’ use of communication strategies affected communication. A multitude of strategies was described, with varying degrees of support. Listening [8, 9, 40, 41, 70], being clear and informative [7, 31, 37–39, 41–43, 59, 68] and facilitating two-way communication [7, 8, 38, 39, 43, 61, 70] were the most supported strategies. Furthermore, different question designs seem to give different perspectives on the patient’s situation and should be used actively.

There appears to be a connection between the use of communication strategies and the other factors linked to the operator: inherent personal characteristics, knowledge and experience. Some strategies could be a natural part of the operator’s personality and therefore part of the operator’s natural communication response, while others might need to be learned through training and education. Personal qualities, such as attitude and emotional control, did impact communication. However, it was a less substantiated factor mentioned only in qualitative studies [9, 26, 27, 31, 32, 36, 41, 45–47, 65].

#### Factors related to the caller

A multitude of methodologically diverse studies described that individual differences between callers affected the conversation [8, 9, 26, 27, 29, 31, 32, 34–40, 42, 43, 45–50, 55–57, 59, 64, 74, 75, 79]. Mismatch between the caller’s expectations and what the service could provide hindered reaching an agreement on measures, which made the communication more difficult [27, 32, 35, 36, 50, 75]. The studies also suggested that callers with lower levels of education and less knowledge were struggling when describing their situation and understanding the information given, which could impede the shared understanding of the situation [27, 47, 56, 74]. Furthermore, the callers’ knowledge and experience affected how the medical problems were presented [6, 8, 9, 26, 27, 31, 32, 34–36, 47, 54, 56, 62, 63, 66, 76–78].

Urgency level and calls regarding mental illness were described in both qualitative and quantitative studies as affecting communication. High-urgency calls were described as streamlined and easy to handle in a qualitative study [8] and of lower quality than less urgent calls in a quantitative study [6]. However, the quality assessment tool used in the latter study did not account for how urgency levels might impact conversation dynamics. Both quantitative and qualitative studies reported that calls regarding mental health symptoms differed from calls regarding somatic symptoms, in being more time-consuming and emotionally demanding [8, 9, 27, 47, 76–78].

#### Factors in the interaction

Factors in the interaction were almost exclusively described in qualitative studies. Faceless communication [27, 31, 32, 34, 35, 39, 44, 47, 52, 58, 59, 65], third-person callers [26–28, 31, 32, 39, 42, 64, 65] and communication barriers [26–28, 31, 32, 34–36, 39, 42, 48, 67, 77] were all supported by a smaller number of studies than many of the other identified factors. The connection between operators and callers was mentioned in many and diverse qualitative studies [7, 9, 26, 27, 31–33, 36, 38, 39, 43, 45–47, 55, 56, 58, 64, 66]. However, both qualitative and quantitative studies described the core element language barriers as hindering good communication [27, 28, 32, 34, 36, 42, 48, 67].

## Discussion

The objective of this study was to identify factors affecting communication during telephone triage and to describe how these factors affected communication. A total of 12 factors were identified to affect the structure, content and flow of communication. The factors were organised into four main themes: organisational factors, factors related to the operator, factors related to the caller and factors in the interaction. All factors were supported by a range of studies (*n* = 9–31). The findings showed that the organisational factors mainly influenced communication by facilitating or complicating the operator’s communication. This suggests that the organisation of the medical call centre has the strongest impact on communication.

### Strengths and limitations

The search strategy was piloted and adjusted ahead of the main search, until known literature was identified in the search result. We chose to search in the main medical databases: the two major medical databases (Medline and Embase) and a database for nursing/allied health professionals (CINAHL). In addition, we supplemented with a search in Web of Science, which is a large interdisciplinary index database. Additional searches in databases such as Scopus might have identified even more studies. However, during the process of analysing the data, it became clear that this material, like other qualitative material, had a saturation point after which no new factors were identified.

The methodological heterogeneity of the studies made the choice of inclusion challenging. Some studies described the factors more subtly than others, and these were often discussed before inclusion. All studies were screened by two or more of the authors, while the synthesis was mainly carried out by one author. Although this approach made the synthesis more consistent, it might also have increased the risk of omitting details of factors or references.

In this review, the data from the quantitative studies was transformed into textual descriptions, to allow for integration with the qualitative data. The transformation might increase the risk of interpretation bias. The methodological heterogeneity in the studies included made the interpretation of the extracted data complex. However, it can be argued this heterogeneity, in which elements are illuminated from different perspectives, strengthens the probability that the phenomenon described is real. Some of the core elements had few references and could have been omitted from the results. We chose to include them, create an overall picture of factors and thereby also describe elements that should be examined more closely. Forty-seven percent of the studies were conducted in Sweden, which could challenge the generalisability of the findings. However, none of the factors identified in this review were only described in studies from one country.

### Implications for further research

The majority of studies included were qualitative studies based on interviews with operators and callers. Interviews shed light on the participants’ experiences and attitudes. While this approach can be used to form hypotheses and models, further research is needed to establish the actual relevance of the identified factors.

A subset of the included studies used conversation analysis or quality measurement tools to assess audio recordings from real conversations. Such methods can capture more of the actual dynamics of the communication process. Employing conversation analysis on audio recordings not only provides a comprehensive understanding of operator-caller interactions, but also allows for a more detailed examination of individual elements in the conversation. However, when using audio recordings, information about the context surrounding the call might be lacking. The results from our review suggest that external factors influence the conversation and need to be considered when analysing audio recordings.

There is an overall lack of studies that explore and substantiate the effects of individual factors. Such studies are needed to balance factors against each other and to gain a deeper understanding of how the factors affect communication and their specific impact on the communication process.

### Implication for practice

**Fig. 5:**
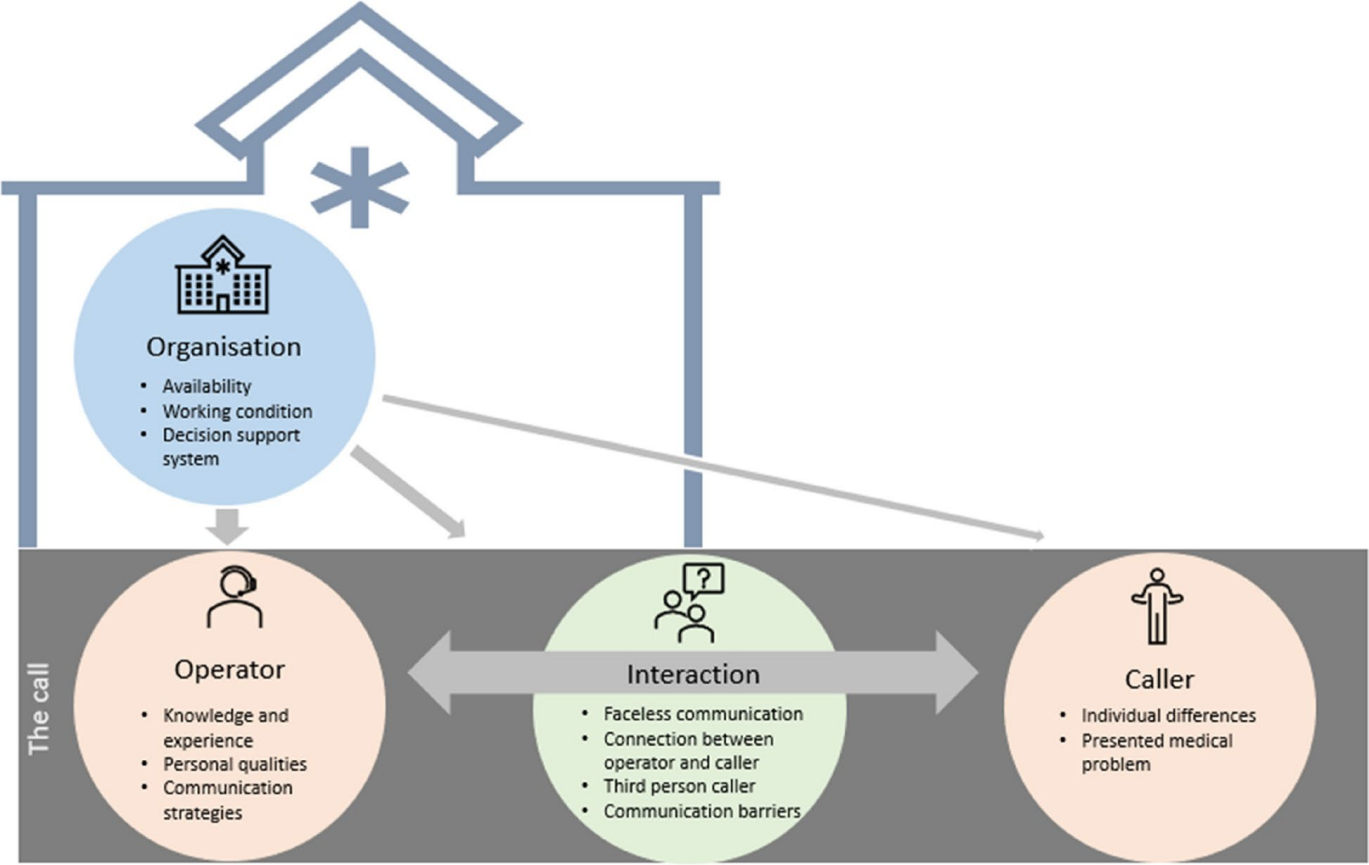
The influence between the organisation, the operator and the caller during communication

Figure 5 shows the influence the organisation, the operator, and the caller has on each other during communication. As parties in the interaction, operators and callers have a direct influence on the communication and thus each other. Nevertheless, the organisation emerged as the most important facilitator of good communication, since availability, working environment and decision support tools had a great influence on how the operator communicates. The operator must rely on the organisation to provide a conducive working environment that promotes communication and learning opportunities, thereby enhancing their competence.

Organisational policies and guidelines are examples of how the organisation influences operators’ communication. Currently, work is being done to standardise and simplify descriptions of symptoms into decision support systems, to ease the assessment of urgency. The organisations argue that standardisation ensures patient safety. Yet this might oversimplify, overlooking the individual differences that need to be considered. Individual differences and differences in how the caller presents the medical problem are well-known challenges in the interaction between healthcare personnel and patients. For decades, researchers have described how patients seek to explain their symptoms by using their own experience, knowledge and surrounding context, and how this affects their health-seeking behaviour, e.g. Leventhal [80]. The use of strict guidelines might challenge the ability to see the overall picture. Additionally, it could discriminate against patients who do not fit the standardisation or fail to describe their symptoms in a way that suit the system. Those who have mental health symptoms, for example, can often initially describe physical symptoms [9]. They need operators with good communication and assessment skills who understand the complexity of the narratives.

Through training and education, the organisation can increase the operator’s communication and assessment skills. Active use of communication strategies seems to be beneficial to communication, but many of the strategies need to be learned. Increased awareness of communication strategies and how they can be utilised can improve the operator’s ability to influence communication.

The organisation can also directly influence the interaction and the caller. One example is the introduction of a video that allows the operator to see the patient, removing some of the barriers to faceless communication. Callers’ expectations and use of the service are influenced by information campaigns [8]. Such campaigns can therefore be used to reduce the mismatch between the callers’ expectations and what the service can provide, and help provide a common starting point for the conversation. In essence, the organisation is the primary driver of enhancing the quality of communication by defining the framework dictating service capabilities and operational procedures.

The dialogue process during telephone triage from an operator’s perspective is well documented and described as containing five phases: opening, listening, analysing, motivating and closing [81]. Still, as found in this review, there are many factors that can complicate the communication, which implies that the operator alone cannot control the communication. There is a need to examine communication from a wider perspective to find what is effective and good communication in medical call centres. The framework developed in this review can be utilised to enhance awareness of the complexity of communication, serving as a foundation for internal quality improvement within the organisation. It can also serve as a starting point for further research.

## Conclusions

Many factors affect the structure, content and flow of communication during telephone triage. The operator influences the quality of communication directly but relies on the organisation to provide a working environment that facilitates good communication. The framework of factors identified in this review can serve as a tool for raising awareness of measures that can improve communication, thereby increasing patient safety. However, the results are mainly based on qualitative studies with a focus on operators’ and callers’ experiences and attitudes. There is limited research into the actual relevance and effect of the identified factors. This needs further investigation.

## Supplementary Information

Additional file 1. PRISMA checklist.

Additional file 2. The search strategy used in Ovid MEDLINE, Embase, Web of Science and Cinahl, respectively.

Additional file 3. Factors identified in the individual studies (*N* = 62).

Additional file 4. Assessment of the methodological limitation of the individual studies (*N* = 62).

## Data Availability

All data generated or analysed during this study are included in this publication.

## Abbreviations

MMAT: Mixed Method Appraisal Tool
PRISMA: Preferred Reporting Items for Systematic Review and Meta-Analyses
DSS: Decision Support System
RICE: Reason for calling; Information gathering; Conclusion; and Evaluation
AQTT: Assessment of Quality in Telephone Triage

## References

[1] Steeman L, Uijen M, Plat E, Huibers L, Smits M, Giesen P. Out-of-hours primary care in 26 European countries: an overview of organizational models. Fam Pract. 2020. 10.1093/fampra/cmaa064. 32597962 10.1093/fampra/cmaa064 PMC7699311

[2] Berchet C, Nader C. The organisation of out-of-hours primary care in OECD countries. OECD Health Working Papers. 2016. 10.1787/5jlr3czbqw23-en.

[3] Lake R, Georgiou A, Li J, Li L, Byrne M, Robinson M, et al. The quality, safety and governance of telephone triage and advice services–an overview of evidence from systematic reviews. BMC Health Serv Res. 2017;17(1):614.4. 28854916 10.1186/s12913-017-2564-x PMC5577663

[4] Midtbø V, Fotland S-LS, Johansen IH, Hunskaar S. From direct attendance to telephone triage in an emergency primary healthcare service: an observational study. BMJ Open. 2022;12(5):e054046. 35501086 10.1136/bmjopen-2021-054046 PMC9062791

[5] Graversen DS, Huibers L, Christensen MB, Bro F, Christensen HC, Vestergaard CH, et al. Communication quality in telephone triage conducted by general practitioners, nurses or physicians: a quasi-experimental study using the AQTT to assess audio-recorded telephone calls to out-of-hours primary care in Denmark. BMJ Open. 2020;10(3):12. 10.1136/bmjopen-2019-033528 PMC7170599 32220912

[6] Huibers L, Keizer E, Giesen P, Grol R, Wensing M. Nurse telephone triage: good quality associated with appropriate decisions. Fam Pract. 2012;29(5):547–52. 22327415 10.1093/fampra/cms005

[7] Strom M, Marklund B, Hildingh C. Callers’ perceptions of receiving advice via a medical care help line. Scand J Caring Sci. 2009;23(4):682–90. 19807883 10.1111/j.1471-6712.2008.00661.x

[8] Greenberg ME. A comprehensive model of the process of telephone nursing. J Adv Nurs. 2009;65(12):2621–9. 19941546 10.1111/j.1365-2648.2009.05132.x

[9] Bjorkman A, Salzmann-Erikson M. When all other doors are closed: Telenurses’ experiences of encountering care seekers with mental illnesses. Int J Ment Health Nurs. 2018;27(5):1392–400. 29383820 10.1111/inm.12438

[10] Ek B, Svedlund M. Registered nurses’ experiences of their decision-making at an Emergency Medical Dispatch Centre. J Clin Nurs. 2015;24(7–8):1122–31. 25273221 10.1111/jocn.12701

[11] Williams B, Warren S, McKim R, Janzen W. Caller self-care decisions following teletriage advice. J Clin Nurs. 2012;21(7–8):1041–50. 22283747 10.1111/j.1365-2702.2011.03986.x

[12] Graversen D, Pedersen A, Carlsen A, Bro F, Huibers L, Christensen M. Quality of out-of-hours telephone triage by general practitioners and nurses: development and testing of the AQTT–an assessment tool measuring communication, patient safety and efficiency. Scand J Prim Health Care. 2019;37(1):18–29. 30689490 10.1080/02813432.2019.1568712 PMC6454404

[13] Derkx HP, Rethans J-JE, Knottnerus JA, Ram PM. Assessing communication skills of clinical call handlers working at an out-of-hours centre: development of the RICE rating scale. Br J Gen Pract. 2007;57(538):383–7. 17504589 PMC2047013

[14] Smits M, Keizer E, Ram P, Giesen P. Development and testing of the KERNset: an instrument to assess the quality of telephone triage in out-of-hours primary care services. BMC Health Serv Res. 2017;17(1):798. 29197376 10.1186/s12913-017-2686-1 PMC5712191

[15] Derkx HP, Rethans JJ, Maiburg BH, Winkens RA, Muijtjens AM, van Rooij HG, et al. Quality of communication during telephone triage at Dutch out-of-hours centres. Patient Educ Couns. 2009;74(2):174–8. 18845413 10.1016/j.pec.2008.08.002

[16] Muka T, Glisic M, Milic J, Verhoog S, Bohlius J, Bramer W, et al. A 24-step guide on how to design, conduct, and successfully publish a systematic review and meta-analysis in medical research. Eur J Epidemiol. 2020;35(1):49–60. 31720912 10.1007/s10654-019-00576-5

[17] Page MJ, McKenzie JE, Bossuyt PM, Boutron I, Hoffmann TC, Mulrow CD, et al. The PRISMA 2020 statement: an updated guideline for reporting systematic reviews. Int J Surg. 2021;88:105906. 33789826 10.1016/j.ijsu.2021.105906

[18] Cooke A, Smith D, Booth A. Beyond PICO: the SPIDER tool for qualitative evidence synthesis. Qual Health Res. 2012;22(10):1435–43. 22829486 10.1177/1049732312452938

[19] Covidence. https://www.covidence.org/. Accessed 22 Apr 2021.

[20] Hong QN, Pluye P, Bujold M, Wassef M. Convergent and sequential synthesis designs: implications for conducting and reporting systematic reviews of qualitative and quantitative evidence. Syst Rev. 2017;6(1):1–14. 28335799 10.1186/s13643-017-0454-2 PMC5364694

[21] Thomas J, Harden A. Methods for the thematic synthesis of qualitative research in systematic reviews. BMC Med Res Methodol. 2008;8(1):1–10. 18616818 10.1186/1471-2288-8-45 PMC2478656

[22] Thomas J, Graziosi, S, Brunton J, Ghouze Z, O'Driscoll P, Bond M, Koryakina A. EPPI-Reviewer: advanced software for systematic reviews, maps and evidence synthesis.: EPPI Centre, UCL Social Research Institute, University College London; 2023. https://eppi.ioe.ac.uk/cms/Default.aspx?alias=eppi.ioe.ac.uk/cms/er4&. Accessed 12 July 2022.

[23] Hong QN, Fàbregues S, Bartlett G, Boardman F, Cargo M, Dagenais P, et al. The Mixed Methods Appraisal Tool (MMAT) version 2018 for information professionals and researchers. Educ Inf. 2018;34(4):285–91.

[24] Ernesater A, Engstrom M, Winblad U, Rahmqvist M, Holmstrom IK. Telephone nurses’ communication and response to callers’ concern–a mixed methods study. Appl Nurs Res. 2016;29:116–21. 26856500 10.1016/j.apnr.2015.04.012

[25] Gamst-Jensen H, Huibers L, Pedersen K, Christensen EF, Ersboll AK, Lippert FK, et al. Self-rated worry in acute care telephone triage: a mixed-methods study. Br J Gen Pract. 2018;68(668):e197–203. 29440015 10.3399/bjgp18X695021 PMC5819985

[26] Gamst-Jensen H, Lippert FK, Egerod I. Under-triage in telephone consultation is related to non-normative symptom description and interpersonal communication: a mixed methods study. Scand J Trauma Resusc Emerg Med. 2017;25(1):52. 28506282 10.1186/s13049-017-0390-0 PMC5433057

[27] Wahlberg AC, Cedersund E, Wredling R. Telephone nurses’ experience of problems with telephone advice in Sweden. J Clin Nurs. 2003;12(1):37–45. 12519248 10.1046/j.1365-2702.2003.00702.x

[28] Gren C, Egerod I, Linderoth G, Hasselager AB, Frederiksen MS, Folke F, et al. “We can’t do without it”: Parent and call-handler experiences of video triage of children at a medical helpline. PLoS ONE. 2022;17(4):e0266007. 35421109 10.1371/journal.pone.0266007 PMC9009705

[29] Thilsted SL, Egerod I, Lippert FK, Gamst-Jensen H. Relation between illness representation and self-reported degree-of-worry in patients calling out-of-hours services: a mixed-methods study in Copenhagen, Denmark. BMJ Open. 2018;8(9):e020401. 30224387 10.1136/bmjopen-2017-020401 PMC6144483

[30] Wahlberg AC, Wredling R. Telephone advice nursing–callers’ experiences. J Telemed Telecare. 2001;7(5):272–6. 11571081 10.1258/1357633011936525

[31] Yliluoma P, Palonen M. Telenurses’ experiences of interaction with patients and family members: nurse-caller interaction via telephone. Scand J Caring Sci. 2020;34(3):675–83. 31657054 10.1111/scs.12770

[32] Eriksson I, Ek K, Jansson S, Sjostrom U, Larsson M. To feel emotional concern: a qualitative interview study to explore telephone nurses’ experiences of difficult calls. Nurs Open. 2019;6(3):842–8. 31367407 10.1002/nop2.264 PMC6650684

[33] Bjorkman A, Salzmann-Erikson M. The bidirectional mistrust: callers’ online discussions about their experiences of using the national telephone advice service. Internet Res. 2018;28(5):1336–50.

[34] Röing M, Rosenqvist UK, Holmström I. Threats to patient safety in telenursing as revealed in Swedish telenurses’ reflections on their dialogues. Scand J Caring Sci. 2013;27(4):969–76. 23289826 10.1111/scs.12016

[35] Röing M, Holmström IK. Malpractice claims in Swedish telenursing: lessons learned from interviews with telenurses and managers. Nurs Res. 2015;64(1):35–43. 25502059 10.1097/NNR.0000000000000063

[36] Lindberg BH, Rebnord IK, Høye S. Phone triage nurses’ assessment of respiratory tract infections–the tightrope walk between gatekeeping and service providing. A qualitative study. Scand J Prim Health Care. 2021;39(2):139–47. 33792485 10.1080/02813432.2021.1908715 PMC8293966

[37] Holmstrom I, Dall’Alba G. ‘Carer and gatekeeper’ - conflicting demands in nurses’ experiences of telephone advisory services. Scand J Caring Sci. 2002;16(2):142–8. 12000667 10.1046/j.1471-6712.2002.00075.x

[38] O’Cathain A, Goode J, Luff D, Strangleman T, Hanlon G, Greatbatch D. Does NHS Direct empower patients? Soc Sci Med. 2005;61(8):1761–71. 15894416 10.1016/j.socscimed.2005.03.028

[39] Holmstrom IK, Nokkoudenmaki MB, Zukancic S, Sundler AJ. It is important that they care - older persons’ experiences of telephone advice nursing. J Clin Nurs. 2016;25(11–12):1644–53. 26961337 10.1111/jocn.13173

[40] Gustafsson S, Walivaara BM, Gabrielsson S. Patient Satisfaction With Telephone Nursing: A Call for Calm, Clarity, and Competence. J Nurs Care Qual. 2020;35(1):E6–11. 30817416 10.1097/NCQ.0000000000000392

[41] Eriksson I, Wilhsson M, Blom T, Broo W, Larsson M. Telephone nurses’ strategies for managing difficult calls: a qualitative content analysis. Nurs Open. 2020;7(6):1671–9. 33072350 10.1002/nop2.549 PMC7544854

[42] Kaminsky E, Carlsson M, Röing M, Holmström IK. If I didn’t trust Swedish Healthcare Direct, I would never call’–views of making pediatric health calls. Clin Nurs Stud. 2013;1(3):57–69.

[43] Winneby E, Flensner G, Rudolfsson G. Feeling rejected or invited: experiences of persons seeking care advice at the Swedish Healthcare Direct organization. Jpn J Nurs Sci. 2014;11(2):87–93. 24698644 10.1111/jjns.12007

[44] Timpka T, Arborelius E. The primary-care nurse’s dilemmas: a study of knowledge use and need during telephone consultations. J Adv Nurs. 1990;15(12):1457–65. 2283459 10.1111/j.1365-2648.1990.tb01789.x

[45] Holmström IK, Krantz A, Karacagil L, Sundler AJ. Frequent callers in primary health care - a qualitative study with a nursing perspective. J Adv Nurs. 2017;73(3):622–32. 27650484 10.1111/jan.13153

[46] Skogevall S, Holmstrom IK, Kaminsky E, Hakansson E. A survey of telephone nurses’ experiences in their encounters with frequent callers. J Adv Nurs. 2020;76(4):1019–26. 31997365 10.1111/jan.14308

[47] Weir H, Waddington K. Continuities in caring? Emotion work in a NHS Direct call centre. Nurs Inq. 2008;15(1):67–77. 18271792 10.1111/j.1440-1800.2008.00391.x

[48] Holmstrom IK, Gustafsson S, Wesstrom J, Skoglund K. Telephone nurses’ use of a decision support system: an observational study. Nurs Health Sci. 2019;21(4):501–7. 31392832 10.1111/nhs.12632

[49] Murdoch J, Barnes R, Pooler J, Lattimer V, Fletcher E, Campbell JL. The impact of using computer decision-support software in primary care nurse-led telephone triage: interactional dilemmas and conversational consequences. Soc Sci Med. 2015;126:36–47. 25514212 10.1016/j.socscimed.2014.12.013

[50] Ernesater A, Holmstrom I, Engstrom M. Telenurses’ experiences of working with computerized decision support: supporting, inhibiting and quality improving. J Adv Nurs. 2009;65(5):1074–83. 19399984 10.1111/j.1365-2648.2009.04966.x

[51] Tuden DS, Borycki EM, Kushniruk AW. Clinical simulation: evaluating the usability of a health information system in a telenurse call centre. Stud Health Technol Inform. 2017;234:340–5. 28186065

[52] Holmstrom I. Decision aid software programs in telenursing: not used as intended? Experiences of Swedish telenurses. Nurs Health Sci. 2007;9(1):21–6. 10.1111/j.1442-2018.2007.00299.x 17300541

[53] Murdoch J, Barnes R, Pooler J, Lattimer V, Fletcher E, Campbell JL. Question design in nurse-led and GP-led telephone triage for same-day appointment requests: a comparative investigation. BMJ Open. 2014;4(3):e004515. 24598305 10.1136/bmjopen-2013-004515 PMC3948453

[54] Morgan JI, Muskett T. Interactional misalignment in the UK NHS 111 healthcare telephone triage service. Int J Med Inform. 2020;134:104030. 31864097 10.1016/j.ijmedinf.2019.104030

[55] Spek M, van Charldorp TC, Vinck VV, Venekamp RP, Rutten FH, Zwart DL, et al. Displaying concerns within telephone triage conversations of callers with chest discomfort in out-of-hours primary care: a conversation analytic study. Patient Educ Couns. 2023;113:107770. 37150153 10.1016/j.pec.2023.107770

[56] Hakimnia R, Holmström IK, Carlsson M, Höglund AT. Exploring the communication between telenurse and caller—a critical discourse analysis. Int J Qual Stud Health Well-being. 2014;9(1):24255. 24964860 10.3402/qhw.v9.24255 PMC4071305

[57] Richards SH, Pound P, Dickens A, Greco M, Campbell JL. Exploring users’ experiences of accessing out-of-hours primary medical care services. Qual Saf Health Care. 2007;16(6):469–77. 18055893 10.1136/qshc.2006.021501 PMC2653185

[58] Cook EJ, Randhawa G, Large S, Ali N, Chater AM, Guppy A. Satisfaction of using a nurse led telephone helpline among mothers and caregivers of young children. Health Policy Technol. 2016;5(2):113–22.

[59] Pettinari CJ, Jessopp L. “Your ears become your eyes”: managing the absence of visibility in NHS Direct. J Adv Nurs. 2001;36(5):668–75. 11737499 10.1046/j.1365-2648.2001.02031.x

[60] Wahlberg AC, Cedersund E, Wredling R. Bases for assessments made by telephone advice nurses. J Telemed Telecare. 2005;11(8):403–7. 16356314 10.1177/1357633X0501100805

[61] Jensen B, Vardinghus-Nielsen H, Mills EHA, Møller AL, Gnesin F, Zylyftari N, et al. “I just haven’t experienced anything like this before”: a qualitative exploration of callers’ interpretation of experienced conditions in telephone consultations preceding a myocardial infarction. Patient Educ Couns. 2023;109:107643. 36716564 10.1016/j.pec.2023.107643

[62] Jensen B, Vardinghus-Nielsen H, Mills EHA, Møller AL, Gnesin F, Zylyftari N, et al. “Like a rainy weather inside of me”: qualitative content analysis of telephone consultations concerning back pain preceding out-of-hospital cardiac arrest. Int Emerg Nurs. 2022;64:101200. 35926318 10.1016/j.ienj.2022.101200

[63] Erkelens DC, van Charldorp TC, Vinck VV, Wouters LT, Damoiseaux RA, Rutten FH, et al. Interactional implications of either/or-questions during telephone triage of callers with chest discomfort in out-of-hours primary care: a conversation analysis. Patient Educ Couns. 2021;104(2):308–14. 32693956 10.1016/j.pec.2020.07.011

[64] Hoglund AT, Holmstrom I. ‘It’s easier to talk to a woman’. Aspects of gender in Swedish telenursing. J Clin Nurs. 2008;17(22):2979–86. 19012768 10.1111/j.1365-2702.2008.02345.x

[65] Holmstrom I, Hoglund AT. The faceless encounter: ethical dilemmas in telephone nursing. J Clin Nurs. 2007;16(10):1865–71. 17880475 10.1111/j.1365-2702.2007.01839.x

[66] Leppanen V. Power in telephone-advice nursing. Nurs Inq. 2010;17(1):15–26. 20137027 10.1111/j.1440-1800.2009.00480.x

[67] Njeru JW, Damodaran S, North F, Jacobson DJ, Wilson PM, St Sauver JL, et al. Telephone triage utilization among patients with limited English proficiency. BMC Health Serv Res. 2017;17(1):706. 29121920 10.1186/s12913-017-2651-z PMC5679138

[68] Gustafsson S, Martinsson J, Wälivaara BM, Vikman I, Sävenstedt S. Influence of self-care advice on patient satisfaction and healthcare utilization. J Adv Nurs. 2016;72(8):1789–99. 27001441 10.1111/jan.12950

[69] Allan JL, Farquharson B, Johnston DW, Jones MC, Choudhary CJ, Johnston M. Stress in telephone helpline nurses is associated with failures of concentration, attention and memory, and with more conservative referral decisions. Br J Psychol. 2014;105(2):200–13. 24754808 10.1111/bjop.12030

[70] Ernesater A, Engstrom M, Winblad U, Holmstrom IK. A comparison of calls subjected to a malpractice claim versus ‘normal calls’ within the Swedish healthcare direct: a case-control study. BMJ Open. 2014;4(10):e005961. 25280808 10.1136/bmjopen-2014-005961 PMC4187455

[71] Vilstrup E, Graversen DS, Huibers L, Christensen MB, Pedersen AF. Communicative characteristics of general practitioner-led and nurse-led telephone triage at two Danish out-of-hours services: an observational study of 200 recorded calls. BMJ Open. 2019;9(6):e028434. 31230024 10.1136/bmjopen-2018-028434 PMC6596995

[72] Moscato SR, Valanis B, Gullion CM, Tanner C, Shapiro SE, Izumi S. Predictors of patient satisfaction with telephone nursing services. Clin Nurs Res. 2007;16(2):119–37. 17452431 10.1177/1054773806298507

[73] Boutin H, Robichaud P, Valois P, Labrecque M. Impact of a continuing education activity on the quality of telephone interventions by nurses in an adult asthma client base. J Nurs Care Qual. 2006;21(4):335–43. 16985404 10.1097/00001786-200610000-00011

[74] Hagan L, Morin D, Lépine R. Evaluation of telenursing outcomes: satisfaction, self-care practices, and cost savings. Public Health Nurs. 2000;17(4):305–13. 10943779 10.1046/j.1525-1446.2000.00305.x

[75] Giesen P, Ferwerda R, Tijssen R, Mokkink H, Drijver R, van den Bosch W, et al. Safety of telephone triage in general practitioner cooperatives: do triage nurses correctly estimate urgency? BMJ Qual Saf. 2007;16(3):181–4. 10.1136/qshc.2006.018846 PMC2465002 17545343

[76] Giesen P, Mokkink H, Hensing M, van den Bosch W, Grol R. Rude or aggressive patient behavior during out-of-hours GP care: challenges in communication with patients. Patient Educ Couns. 2008;73(2):205–8. 18547777 10.1016/j.pec.2008.04.009

[77] Hansen EH, Hunskaar S. Understanding of and adherence to advice after telephone counselling by nurse: a survey among callers to a primary emergency out-of-hours service in Norway. Scand J Trauma Resusc Emerg Med. 2011;19(1):48. 21892945 10.1186/1757-7241-19-48 PMC3177778

[78] Leclerc B, Dunnigan L, Côté H, Zunzunegui M, Hagan L, Morin D, et al. Callers’ ability to understand advice received from a telephone health-line service: comparison of self-reported and registered data. Health Serv Res. 2003;38(2):697–710. 12785568 10.1111/1475-6773.00140 PMC1360910

[79] Strom M, Baigi A, Hildingh C, Mattsson B, Marklund B. Patient care encounters with the MCHL: a questionnaire study. Scand J Caring Sci. 2011;25(3):517–24. 21338380 10.1111/j.1471-6712.2010.00858.x

[80] Leventhal H. The common sense representation of illness danger. Contrib Med Psychol. 1980;2:7.

[81] Gustafsson SR, Wahlberg AC. The telephone nursing dialogue process: an integrative review. BMC Nurs. 2023;22(1):345. 10.1186/s12912-023-01509-0. 37770869 10.1186/s12912-023-01509-0 PMC10537534

