# Correction: Factors affecting communication during telephone triage in medical call centres: a mixed methods systematic review

**DOI:** 10.1186/s13643-026-03084-2

**Published:** 2026-02-07

**Authors:** Siri‑Linn Schmidt Fotland, Vivian Midtbø, Jorunn Vik, Erik Zakariassen, Ingrid Hjulstad Johansen

**Affiliations:** 1 National Centre for Emergency Primary Health Care, NORCE Norwegian Research Centre AS, Box 22, Bergen, NO‑5838 Norway; 2 Department of Global Public Health and Primary Care, Faculty of Medicine, University of Bergen, Box 7804, Bergen, NO‑5020 Norway; 3 The Regional Centre for Emergency Medical Research and Development in Western Norway (RAKOS), Stavanger University Hospital, Box 8100, Stavanger, NO‑4068 Norway

**Correction: Syst Rev 13, 162 (2024)**

10.1186/s13643-024-02580-7

Following publication of the original article [1], the authors reported that three paragraphs are missing from the section *Qualitative findings*, under the subheading *Factors related to the caller,* after *Individual differences*. The missing section is written below.

**Presented medical problem**

Callers’ interpretations of their symptoms were based on knowledge and previous experience and affected their choice of words when describing symptoms [29, 61, 66]. When callers were uncertain of which symptom or observation that was important, or lacked knowledge, terminology, or ability to succinctly describe the symptom, they often presented more information than necessary, or gave a vague or wrong description [31, 32, 34, 54, 56, 63]. Given their experience with callers who could both exaggerate and downplay their symptoms, operators described an underlying skepticism toward callers’ descriptions that complicated interpretation of the symptoms [27, 32, 36]. Another obstacle described was when callers themselves presented a diagnosis or solution, and the operators chose to draw conclusions based solely on this information [35].

The level of urgency of the medical situation affected the communication. A conversation with high acuity was characterised as more streamlined, straightforward, and easy to handle, due to the need for measures to be initiated quickly [8].

Operators described calls regarding mental illness as different from calls regarding somatic illness [8, 9, 27, 47]. Due to shame, callers often hid the true reason for their call behind diffuse symptoms [9]. Conversations dealing with mental illness were described as difficult, time-consuming, requiring multiple nursing skills, exhausting, and emotionally demanding for the operators, which made the operators more reluctant towards these conversations [8, 9, 27, 47].

The original article [1] has been corrected.
