# Supplementary material for: Factors affecting communication during telephone triage in medical call centres: a mixed methods systematic review
Source: Syst Rev. 2024 Jun 22;13:162. doi: 10.1186/s13643-024-02580-7 (PMC11193260; doi:10.1186/s13643-024-02580-7)
Supplement: Supplementary file 2 — Additional file 2. The search strategy used in Ovid MEDLINE, Embase, Web of Science and Cinahl, respectively. [file 13643_2024_2580_MOESM2_ESM.docx]

**Additional file 2: The search strategy used in Ovid MEDLINE, Embase, Web of Science and Cinahl, respectively**

**Ovid MEDLINE(R) and Epub Ahead of Print, In-Process, In-Data-Review & Other Non-Indexed Citations and Daily <1946 to June 28, 2023>**

Date: 29.06.2023

1 Triage/ or triage*.ti,ab,kf. 31537

2 exp Telephone/ or telephone*.ti,ab,kf. 96238

3 1 and 2 1611

4 ((Telephone or remote) adj3 (triage* or under-triage* or undertriage*)).ti,ab,kf. 835

5 Telenursing/ or telenurs*.ti,ab,kf. 485

6 emergency medical dispatch/ or hotlines/ or Emergency Medical Services/ 51443

7 (emergency medical dispatch or hotline* or emergency medical service* or emergency call center* or emergency medical communication center*).ti,ab,kf. 14586

8 exp After-Hours Care/ or (after hours care or out of hours care or out of hours medical care).ti,ab,kf. 2404

9 Gatekeeping/ or gatekeeping.ti,ab,kf. 1544

10 Remote Consultation/ or (remote consult* or teleconsult* or telephone consult*).ti,ab,kf. 8795

11 or/5-10 70398

12 11 and 2 5276

13 3 or 4 or 12 6299

14 exp Decision Making/ or decision making.ti,ab,kf. 376873

15 Nursing Assessment/ or nursing assessment*.ti,ab,kf. 30120

16 exp Communication/ or (communication or communication barrier* or language barrier*).ti,ab,kf. 608329

17 nurse-patient relations/ or physician-patient relations/ 111142

18 ((nurse-patient or physician-patient) adj3 (relation* or communication or conversation*)).ti,ab,kf. 7466

19 Symptom Assessment/ or (symptom adj3 (assessment* or evaluation*)).ti,ab,kf. 13817

20 ((interpersonal or interactional) adj3 (communication or conversation* or dilemma*)).ti,ab,kf. 3848

21 ((conversation* or communication*) adj3 consequence*).ti,ab,kf. 306

22 or/14-21 1061738

**23 13 and 22 1358**

<https://ovidsp.ovid.com/ovidweb.cgi?T=JS&NEWS=N&PAGE=main&SHAREDSEARCHID=15558Kec8M0alaolvWC6kJPHAgb9ET6aBOr07EW8uC1jcD9Dla713BcczcqtqbaEz>

**Embase <1974 to 2023 June 28>**

Date: 29.06.2023

1 emergency health service/ or emergency medical dispatch/ or hotline/ 117848

2 (emergency medical dispatch or hotline* or emergency medical service* or emergency call center* or emergency medical communication center*).ti,ab,kw. 20558

3 telenursing/ or telenurs*.ti,ab,kw. 566

4 teleconsultation/ or (remote consult* or teleconsult* or telephone consult*).ti,ab,kw. 17488

5 telephone/ or telephone*.ti,ab,kw. 110884

6 triage*.ti,ab,kw. 42258

7 5 and 6 2200

8 1 or 2 or 3 or 4 140899

9 5 and 8 10564

10 ((Telephone or remote) adj3 (triage* or under-triage* or undertriage*)).ti,ab,kw. 1179

11 7 or 9 or 10 11240

12 exp decision making/ or decision making.ti,ab,kw. 556315

13 nursing assessment/ or nursing assessment*.ti,ab,kw. 28504

14 exp interpersonal communication/ or (communication or language).ti,ab,kw. 1160734

15 ((interpersonal or interactional) adj3 (communication or conversation* or dilemma*)).ti,ab,kw. 3950

16 ((conversation* or communication*) adj3 (consequence* or implication*)).ti,ab,kw. 1835

17 nurse patient relationship/ or doctor patient relationship/ 41880

18 ((nurse-patient or physician-patient) adj3 (relation* or communication or conversation*)).ti,ab,kw. 5749

19 symptom assessment/ or (symptom adj3 (assessment* or evaluation*)).ti,ab,kw. 20483

20 12 or 13 or 14 or 15 or 16 or 17 or 18 or 19 1730478

**21 11 and 20 2395**

<https://ovidsp.ovid.com/ovidweb.cgi?T=JS&NEWS=N&PAGE=main&SHAREDSEARCHID=4HQ0DmtUL7yP1eu2MLymBtqUA5Y3gubZ1VK8eaFkJSHTMgKErabTS6RoxK2Wnvrbd>

**Web of Science Core Collection**

Date: 29.06.2023

Entitlements/editions:

- WOS.SCI: 1945 to 2023

- WOS.AHCI: 1975 to 2023

- WOS.ESCI: 2018 to 2023

- WOS.SSCI: 1956 to 2023

Searches:

1: TS=(((Triage* AND telephone) ) )

Results: 1389

2: TS=( (((telephone OR remote) NEAR/3 (triage* OR under-triage* OR undertriage*) )) )

Results: 955

3: #1 OR #2

Results: 1476

4: TS=(((emergency NEAR/3 (dispatch OR service* OR center*) )) )

Results: 31172

5: TS=(((("after hours" OR "out of hours") NEAR/2 care)) )

Results: 879

6: TS=(((gatekeeping OR hotline* OR telenursing) ) )

Results: 4281

7: TS=((("remote consult*" OR teleconsult* OR "telephone consult*") ) ) Results: 4276

8: TS=((telephone) ) Results: 80789

9: #7 OR #6 OR #5 OR #4 Results: 40210

10: #9 AND #8 Results: 2935

11: #10 OR #3 Results: 3969

12: TS=(("decision making") ) Results: 425392

13: TS=(("nursing assessment*") ) Results: 1246

14: TS=(((communication OR language) ) ) Results: 1422865

15: TS=(((("Nurse-patient" OR "physician-patient") NEAR/3 (relation* OR communication* OR conversation*) )) ) Results: 6483

16: TS=(((symptom NEAR/3 (assessment* OR evaluation*) )) ) Results: 20821

17: TS=((((interpersonal OR interactional) NEAR/3 (communication OR conversation* OR dilemma*) )) )

Results: 6482

18: TS=((((conversation* OR communication*) NEAR/3 (consequence* OR implication*) )) )

Results: 4178

19: #18 OR #17 OR #16 OR #15 OR #14 OR #13 OR #12

Results: 1834337

**20: #19 AND #11**

**Results: 730**

[**https://www.webofscience.com/wos/woscc/summary/69a4b762-ee8a-4014-ac97-e7e66330fac6-94b3d828/relevance/1**](https://www.webofscience.com/wos/woscc/summary/69a4b762-ee8a-4014-ac97-e7e66330fac6-94b3d828/relevance/1)

**Cinahl**

Date: 29.06.2023

Interface - EBSCOhost Research Databases
Search Screen - Advanced Search
Database – CINAHL

Expanders - Apply equivalent subjects
Search modes - Boolean/Phrase

| **#** | **Query** | **Results** |
| --- | --- | --- |
| S24 | S14 AND S23 | 2,130 |
| S23 | S15 OR S16 OR S17 OR S18 OR S19 OR S20 OR S21 OR S22 | 1,010,611 |
| S22 | TX ((conversation* OR communication*) N3 (consequence* OR implication*)) | 1,492 |
| S21 | ((Interpersonal OR interactional) N3 (communication OR conversation OR dilemma*)) | 2,084 |
| S20 | (symptom N3 (assessment* OR evaluation*) | 8,544 |
| S19 | TX (("nurse-patient" OR "physician-patient") N3 (relation* OR communication OR conversation)) | 64,075 |
| S18 | (MH "Physician-Patient Relations") OR (MH "Nurse-Patient Relations") | 62,916 |
| S17 | (MH "Communication+") OR TX ( (communication OR language) ) | 677,657 |
| S16 | (MH "Nursing Assessment") OR TX "nursing assessment*" | 19,610 |
| S15 | ( (MH "Decision Making") OR (MH "Decision Making, Clinical") ) OR TX decision making | 311,011 |
| S14 | S3 OR S4 OR S13 | 6,516 |
| S13 | S2 AND S12 | 6,059 |
| S12 | S5 OR S6 OR S7 OR S8 OR S9 OR S10 OR S11 | 147,406 |
| S11 | (MH "Remote Consultation") OR TX ( (remote consult* OR teleconsult* OR telephone consult*) ) | 4,730 |
| S10 | (MH "Gatekeeping") OR TX gatekeeping | 1,004 |
| S9 | ("after hours care" OR "out of hours care") | 282 |
| S8 | TX hotline* | 1,970 |
| S7 | TX (emergency N3 (dispatch OR service* OR center*)) | 115,873 |
| S6 | (MH "Emergency Medical Services+") | 116,407 |
| S5 | (MH "Telenursing") OR telenurs* | 2,377 |
| S4 | TX ((telephone OR remote) N3 (triage* OR under-triage* OR undertriage*)) | 875 |
| S3 | S1 AND S2 | 1,610 |
| S2 | (MH "Telephone+") OR TX telephone* | 62,334 |
| S1 | (MH "Triage") OR TX triage* | 19,525 |

[**Cinahl search**](https://search.ebscohost.com/login.aspx?direct=true&db=cin20&bquery=((((MH+%26quot%3bTriage%26quot%3b)+OR+TX+triage*)+AND+((MH+%26quot%3bTelephone%2b%26quot%3b)+OR+TX+telephone*))+OR+(TX+((telephone+OR+remote)+N3+(triage*+OR+under-triage*+OR+undertriage*)))+OR+(((MH+%26quot%3bTelephone%2b%26quot%3b)+OR+TX+telephone*)+AND+(((MH+%26quot%3bTelenursing%26quot%3b)+OR+telenurs*)+OR+((MH+%26quot%3bEmergency+Medical+Services%2b%26quot%3b))+OR+(TX+(emergency+N3+(dispatch+OR+service*+OR+center*)))+OR+(TX+hotline*)+OR+((%26quot%3bafter+hours+care%26quot%3b+OR+%26quot%3bout+of+hours+care%26quot%3b))+OR+((MH+%26quot%3bGatekeeping%26quot%3b)+OR+TX+gatekeeping)+OR+((MH+%26quot%3bRemote+Consultation%26quot%3b)+OR+TX+((remote+consult*+OR+teleconsult*+OR+telephone+consult*))))))+AND+((((MH+%26quot%3bDecision+Making%26quot%3b)+OR+(MH+%26quot%3bDecision+Making%2c+Clinical%26quot%3b))+OR+TX+decision+making)+OR+((MH+%26quot%3bNursing+Assessment%26quot%3b)+OR+TX+%26quot%3bnursing+assessment*%26quot%3b)+OR+((MH+%26quot%3bCommunication%2b%26quot%3b)+OR+TX+((communication+OR+language)))+OR+((MH+%26quot%3bPhysician-Patient+Relations%26quot%3b)+OR+(MH+%26quot%3bNurse-Patient+Relations%26quot%3b))+OR+(TX+((%26quot%3bnurse-patient%26quot%3b+OR+%26quot%3bphysician-patient%26quot%3b)+N3+(relation*+OR+communication+OR+conversation)))+OR+(symptom+N3+(assessment*+OR+evaluation*))+OR+(((Interpersonal+OR+interactional)+N3+(communication+OR+conversation+OR+dilemma*)))+OR+(TX+((conversation*+OR+communication*)+N3+(consequence*+OR+implication*))))&type=1&searchMode=Standard&site=ehost-live)
